# Estimating the impact of Tiny Targets in reducing the incidence of Gambian sleeping sickness in the North-west Uganda focus

**DOI:** 10.1186/s13071-021-04889-x

**Published:** 2021-08-18

**Authors:** Paul R. Bessell, Johan Esterhuizen, Michael J. Lehane, Joshua Longbottom, Albert Mugenyi, Richard Selby, Inaki Tirados, Steve J. Torr, Charles Waiswa, Charles Wamboga, Andrew Hope

**Affiliations:** 1 Independent Consultant, Pencaitland, Scotland, UK; 2 Liverpool School of Tropical Medicine (LSTM), Pembroke Place, Liverpool, UK; 3 Coordinating Office for Control of Trypanosomiasis in Uganda (COCTU), Kampala, Uganda; 4 Ministry of Health (MOH), Kampala, Uganda

**Keywords:** Human African trypanosomiasis, Tsetse control, Tiny Targets, Uganda, Disease control, Elimination

## Abstract

**Background:**

Riverine species of tsetse (*Glossina*) transmit *Trypanosoma brucei gambiense*, which causes Gambian human African trypanosomiasis (gHAT), a neglected tropical disease. Uganda aims to eliminate gHAT as a public health problem through detection and treatment of human cases and vector control. The latter is being achieved through the deployment of ‘Tiny Targets’, insecticide-impregnated panels of material which attract and kill tsetse. We analysed the spatial and temporal distribution of cases of gHAT in Uganda during the period 2010–2019 to assess whether Tiny Targets have had an impact on disease incidence.

**Methods:**

To quantify the deployment of Tiny Targets, we mapped the rivers and their associated watersheds in the intervention area. We then categorised each of these on a scale of 0–3 according to whether Tiny Targets were absent (0), present only in neighbouring watersheds (1), present in the watersheds but not all neighbours (2), or present in the watershed and all neighbours (3). We overlaid all cases that were diagnosed between 2000 and 2020 and assessed whether the probability of finding cases in a watershed changed following the deployment of targets. We also estimated the number of cases averted through tsetse control.

**Results:**

We found that following the deployment of Tiny Targets in a watershed, there were fewer cases of HAT, with a sampled error probability of 0.007. We estimate that during the intervention period 2012–2019 we should have expected 48 cases (95% confidence intervals = 40–57) compared to the 36 cases observed. The results are robust to a range of sensitivity analyses.

**Conclusions:**

Tiny Targets have reduced the incidence of gHAT by 25% in north-western Uganda.

**Graphical abstract:**

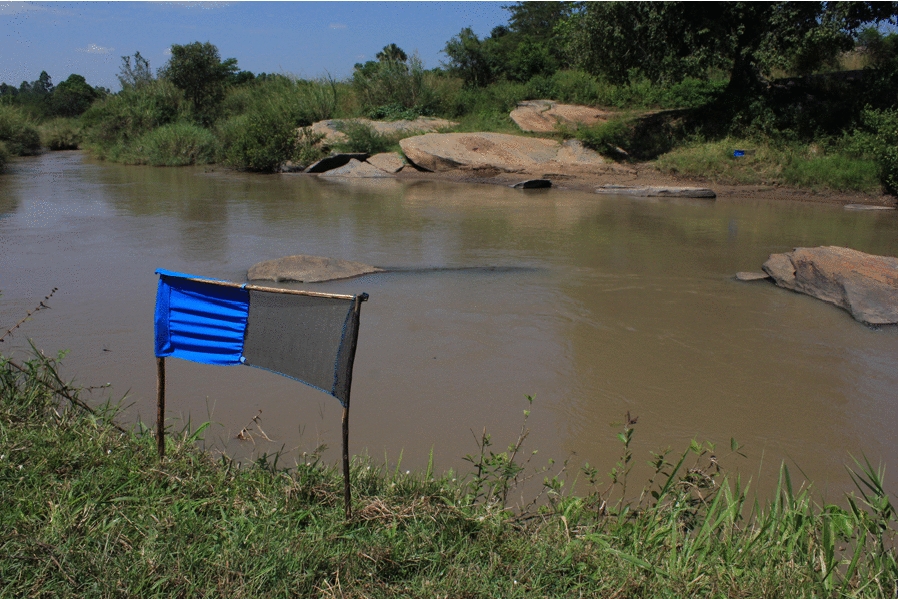

**Supplementary Information:**

The online version contains supplementary material available at 10.1186/s13071-021-04889-x.

## Background

Human African trypanosomiasis (HAT) is a fatal neglected tropical disease that occurs in rural areas of sub-Saharan Africa. The disease exists in two forms, Gambian HAT (gHAT), caused by *Trypanosoma brucei gambiense*, and Rhodesian HAT (rHAT), caused by *T. b. rhodesiense*; both forms are transmitted by tsetse flies (*Glossina* spp.). The Gambian form of the disease is found in West and Central Africa and is transmitted by riverine species of tsetse; it causes a chronic infection and is responsible for greater than 95% of HAT cases reported annually [1]. The Rhodesian form is an acute infection which occurs in East and Southern Africa where it is transmitted by savanna species of tsetse. Uganda is the only country in the world with both forms of the disease; however, there is no known geographical overlap of disease transmission.

The first half of the twentieth century saw devastating epidemics of HAT including one epidemic in Uganda which is estimated to have killed more than 200,000 people [2]. Large-scale screening and treatment campaigns successfully brought the numbers of cases down so that by the mid-1960s the disease was close to elimination. This low endemicity led to a shift in priorities, and as a consequence there was a resurgence of the disease [3]. By 1998 the global number of reported gHAT cases was greater than 37,000, but estimates suggest that 300,000 cases were either missed or misdiagnosed and therefore were not treated [3]. Renewed efforts to control the disease saw the number of cases decline again, and by 2009 fewer than 10,000 cases were reported annually [1, 4]. The disease is targeted for elimination of transmission by 2030 [5].

The mainstay of gHAT control has historically been mass screening and treatment of populations with little involvement of vector control, as the available methods were considered to not be cost-effective and were logistically challenging to implement. However, as the prevalence of HAT decreases, the cost-effectiveness of mass active screening for HAT decreases exponentially [6]. This can be addressed through passive screening [7] and through vector control with Tiny Targets, a novel, cost-effective technology comprising insecticide-treated panels of material which attract and kill riverine tsetse [8–11]. Tiny Targets have been shown to reduce tsetse fly densities by more than 80% in Chad [12, 13], Côte d’Ivoire [14], the Democratic Republic of the Congo (DRC) [15] and Uganda [9], but by a lower amount in Guinea, where the deployment methodology is different [16]. In Guinea and Chad, the use of Tiny Targets to reduce transmission has been shown to reduce the incidence of gHAT cases [12, 17]. In the DRC, which is the country that contributes the greatest number of cases, modelling studies have demonstrated that in some foci of the DRC, relying on screening and treatment alone will not be sufficient to achieve elimination goals on time, but that the addition of vector control will accelerate progress [18].

In Uganda, the historical gHAT foci are in the West Nile region in the north-west of the country, where the disease is transmitted by *Glossina fuscipes fuscipes*. In 2011, Tiny Targets were introduced in two districts as a trial to assess the feasibility and effectiveness of using the technology at scale [9]. The success of the trial led to the scaling up into full tsetse control in 2014 to contribute to the gHAT elimination effort in Uganda, with Tiny Targets deployed in five districts. Tsetse control efforts subsequently expanded in 2017 from five to seven districts covering around 3900 km^2^ [19]. As a consequence of these measures, tsetse densities across the region have been reduced by more than 90% [9]. Here we quantify the impact of vector control on the spatial and temporal distribution of cases and infer the impact on disease incidence [9].

## Methods

### Study area

**Fig. 1:**
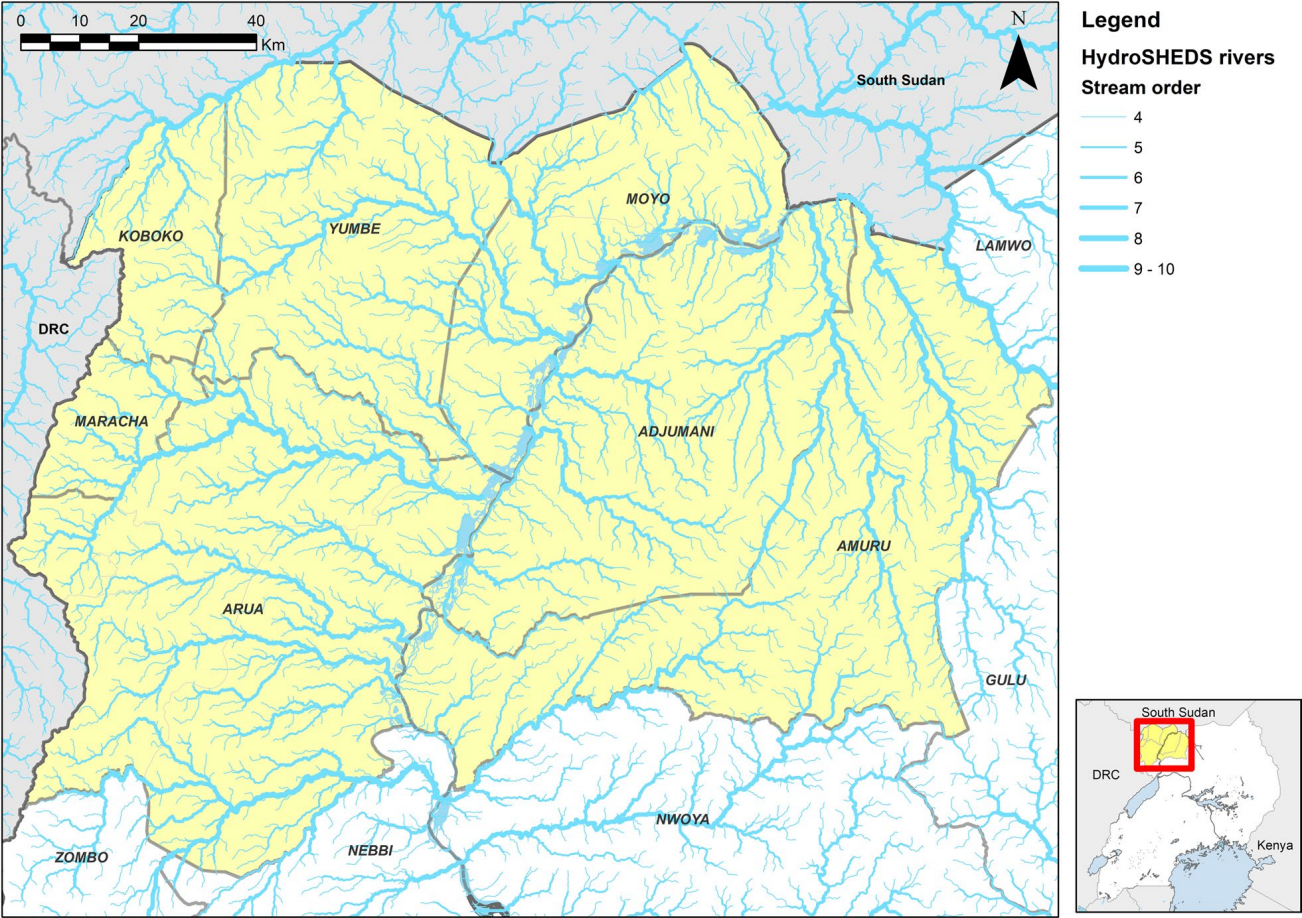
Map showing the study area (in yellow) and the rivers as extracted by HydroSHEDS created from the NASA SRTM1 DEM using ESRI ArcGIS 10.5

The study was conducted in the gHAT endemic area of north-west Uganda in the districts of Adjumani, Amuru, Arua, Koboko, Maracha, Moyo and Yumbe [7] (Fig. 1).

### Geographical data processing

In Uganda, Tiny Targets are deployed along the larger permanent rivers and streams. However, to define an intervention area, it is necessary to define an area around the watersheds that is controlled, and so to do this we identified the watersheds that had been controlled. To identify watersheds, we used NASA Shuttle Radar Topography Mission (SRTM) digital elevation model (DEM) imagery, produced at a spatial resolution of 1 arcsecond (~ 30 m × 30 m) [20], to extract HydroSHEDS [21] in ESRI ArcGIS (v10.5). The HyrdoSHEDS method identifies rivers and streams and orders them according to their number of tributaries (Fig. 1), so an order 1 river has no tributaries, order 2 has one or more order 1 tributaries, etc.

**Fig. 2:**
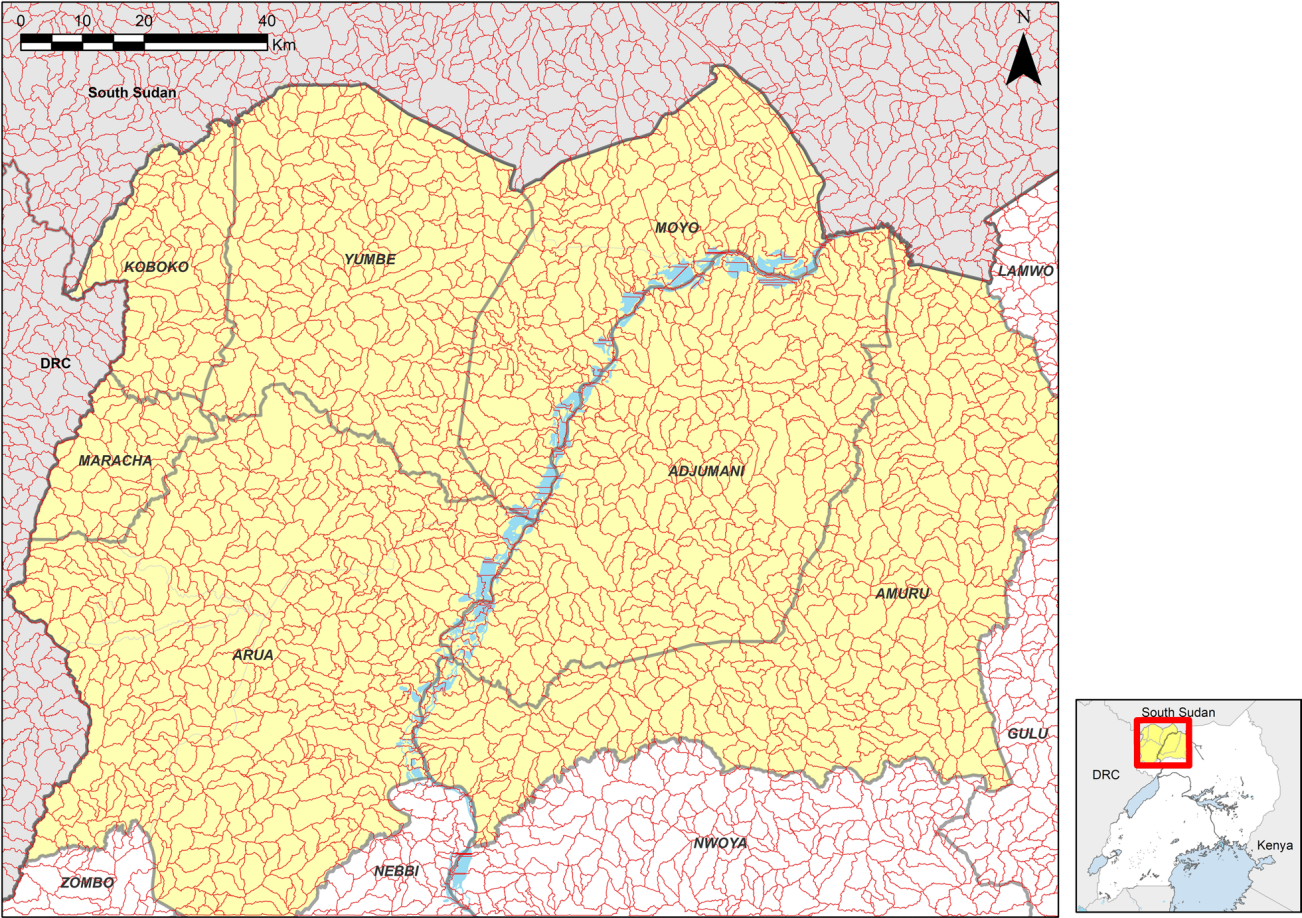
Watersheds for the study area in red; each of the polygons represents a single watershed derived from HydroSHEDS created from the NASA SRTM1 DEM using ESRI ArcGIS 10.5

Watersheds are defined as the area of land that drains into a single water body. To define watersheds, we extracted all order 4 or greater rivers (rivers with two or more third-order tributaries [22]), and from these identified pour points at the intersections of rivers. Pour points define the end point of the watershed. The pour points were used to define watersheds using the methodology described by ESRI ArcGIS (support article 000012346). The resulting watersheds for the area are shown in Fig. 2 (mean area 9.8km^2^, range 1.02–57.4 km^2^). The resulting number of watersheds that are defined exceeds the number of rivers that were controlled using Tiny Targets, as the defined watersheds include some non-permanent streams, or simply drainage features that do not have any sort of flowing water. Consequently, the density of watersheds is typically greater than the density of rivers defined by Tiny Target deployments.

### Tiny Target deployment

**Fig. 3:**
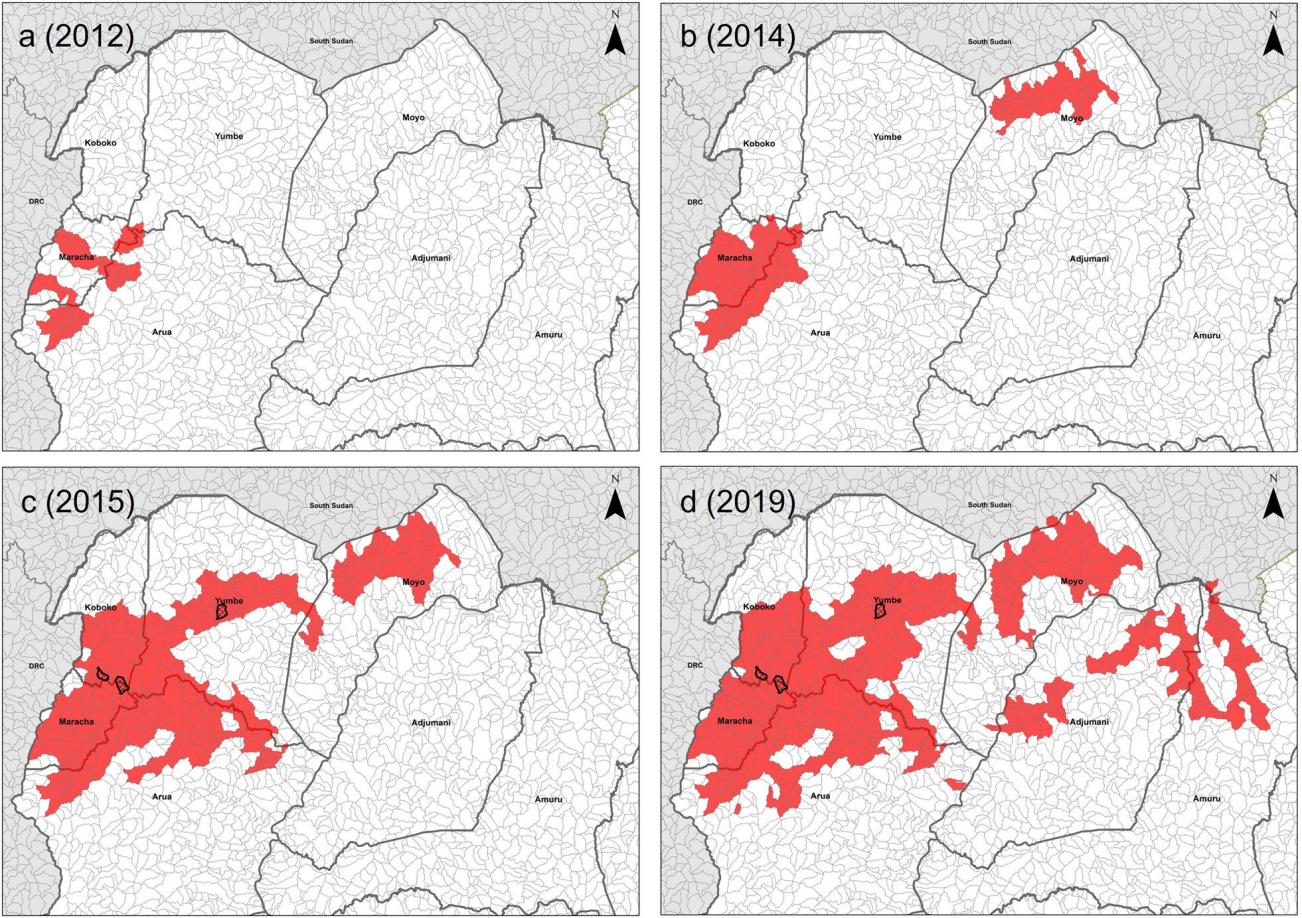
Map of watersheds deployed for four key intervention points. The black outlines represent the island watersheds that were filled in

We used Tiny Target deployment data in Uganda between December 2011 and 2019, conducted at approximately 6-month intervals, giving a total of 15 deployments. For each deployment, the locations of targets were recorded using global positioning systems. For the first two deployments (2011–2012), Tiny Targets were deployed in five separate 7 × 7 km blocks [9]. Thereafter (2013–2019), deployments were along continuous stretches of river. The coverage of each deployment was overlain onto the watershed polygons to identify polygons where targets were present. For the first two deployments (2011–2012), watershed polygons were regarded as treated if they had at least 500 m of river that was deployed in the watershed area. Thereafter, a polygon was considered controlled if targets were deployed on at least two occasions during 2011 to 2019, with control being deemed to have started at the earliest deployment. For nearly all (98%) deployed areas, once an area was deployed, it was deployed in all subsequent years. If deployment was between January and June, then deployment was deemed to have started that year. If deployment first occurred in the second half of the year, control was deemed to have started at the beginning of the next year. The rationale behind this is that the reduction in tsetse numbers following deployment of Tiny Targets is gradual and takes several months to reach reductions of up to 90%. Thus, for each year between 2012 (the deployment in 2011 was in the second half of the year, so defined as 2012) and 2019 we are able to identify where a watershed had a deployment in that year (Fig. 3). As our definition of watersheds includes polygons that are smaller than the deployed rivers, we identified watersheds that did not contain a deployment but were surrounded entirely by polygons containing deployments. Such polygons were marked as controlled for the purpose of analysis, as it could be assumed that there was no permanent drainage feature there or it would be controlled by local dispersal of tsetse [22].

To allow for the mobility of tsetse and humans, we classified each watershed polygon in each year on a scale of 0 to 3 according to the presence/absence of targets in neighbouring polygons (Table 1, Additional file 1: Figures S1–S8).

**Table 1:** Table to describe the deployment zone score classification system based on the deployment status of the watershed and its neighbours

Classification score	Watershed	Neighbouring watersheds
0	Not deployed	No neighbours are deployed
1	Not deployed	One or more neighbours are deployed
2	Deployed	One or more neighbours are not deployed
3	Deployed	All neighbours are deployed

### HAT case data

HAT case data were obtained from the WHO HAT Atlas for the period 2000–2011 [23] and from the Ugandan Ministry of Health through the Trypa-NO! Programme for the years 2012–2020. Data from the WHO HAT Atlas provided point geolocations for 4165 cases between 2000 and 2011, but 30 cases were excluded because they could not be georeferenced. The Ugandan Ministry of Health supplied data on a further 56 cases from 2012–2020. Data from 2012–2020 were geolocated by mapping the villages and cross-checking publicly available data sets, principally OpenStreetMap, Google Maps and GeoNames [19]. Where these were not able to provide a location for a village, the case was mapped to the school (the school being an identifiable place reflective of the centre of population) of the patient’s parish. Four of the cases that were reported since 2016 were identified as being refugees from South Sudan; it is estimated that > 650,000 refugees from South Sudan have settled in northern Uganda since 2016 (https://data2.unhcr.org/en/situations/southsudan/location/1925). South Sudan is a gHAT focus [24]; the provenance of infection of these cases cannot be determined with certainty, and so the cases were excluded from further analysis. A further case of a Ugandan who was resident in South Sudan was also excluded. This gives a total of 4186 georeferenced cases between 2000 and 2020.

### Data analysis

If Tiny Targets reduce the incidence of gHAT, then the incidence of cases will be lower in areas where Tiny Targets were continuously deployed. Accordingly, we proposed the following hypothesis:

- Null hypotheses (*H*_0_) = The number of cases in a polygon was not significantly affected by the deployment of targets in the polygon and/or its neighbouring polygons.
- Alternative hypothesis (*H*_1_) = Following the deployment of Tiny Targets in a polygon and/or its neighbours, the relative number of cases within that area declined.

For each year with vector control, we define two populations of cases by overlaying the case locations onto the deployment polygons:

1. *Cases* are gHAT cases in the north-west Uganda focus that were reported following the start of the Tiny Target intervention (taken as 2012, as the intervention commenced in December 2011) and broken down by the year in which we assume that they were infected. The date of infection we infer from the reporting date. For stage 1 cases, we conservatively assume that the infection date is the same as the reporting date. For stage 2 cases, we assume that the case was infected 263 days before the reporting date, this being the lower limit of the duration of stage 1 in passive screening from Checchi et al. [25].
2. *Case–controls* are all gHAT cases that were infected between 2000 and 2020.

Using these categories, we conduct two analyses: one on the impact of the deployments, and the second on the number of cases prevented.

### Deployment impact analysis

For each year from the period 2012–2020, the cases and matched case–controls are overlain onto the vector control deployment zones for that year, and we determine the vector control zone class score (Table 1) for the location of each case and each case–control. Thus, starting with 2012, we take the cases with a putative infection date in that year and extract the deployment scores for those cases. As the comparison controls, we extract the deployment scores for all cases between 2000 and 2020 and randomly sample a number of controls equal to the number of cases for that year.

The above procedure was implemented in R version 3.6.0 [26] with the following algorithm:

1. Starting with 2012 as the analysis year:
  - Take the cases that were estimated to have been infected in that year.
  - From the case–controls, we randomly sample a number equal to the number of cases for that year. So, if there are four cases in a year, then we randomly sample four from the 4187 case–controls for 2000–2020 and extract their deployment score based on where it would be relative to the deployments in 2012 (and then for subsequent years).
  - We sum the deployment zone scores for both the cases and the sampled case–controls.
2. Repeat a–c for each year (i.e. 2013, 2014…2019).
3. Across the years we sum the scores for the cases versus the sampled case–controls. If the total deployment score for the case–controls is lower than that for the cases, that would suggest that the cases are more likely to be in the deployment area compared to the overall distribution of cases. If the scores of the case–controls are greater than those for the cases, then the cases are less likely to be in the deployment area than would be expected.
4. Repeat steps 1–3 for 1,000,000 replicates.
5. The data are analysed by the proportion of iterations for which the cases had a higher score compared to the sampled case–controls. A proportion below 5% would represent statistical significance at the 95% level.

### Cases prevented analysis

We also estimate the number of cases that were averted by the intervention. This is based on the premise that there is a case incidence rate in the controlled areas and a separate case incidence rate in the uncontrolled area. For example, suppose that prior to control there may have been an incidence rate of 4/10,000 inside the controlled area and 2/10,000 outside, so the controlled area has twice the incidence rate of the uncontrolled area. We can continue to measure the incidence rate of the uncontrolled area following the start of the intervention, and if the intervention has reduced the incidence rate, then there should be a drop in the incidence rate in the controlled area relative to the uncontrolled area. So, if following the start of the intervention the incidence rate outside the controlled area fell from 2/10,000 to 1/10,000 and the incidence rate inside the controlled area fell from 4/10,000 to 2/10,000, this would indicate that the control had no impact on case numbers, but a fall in the controlled area to 1/10,000 would indicate a 50% drop in the incidence rate as a result of the intervention. Thus, we can use this relative difference to estimate the number of cases that were prevented in the controlled area by the vector control measures.

To carry out this analysis we must invert the deployment zone scores so that a score of 3 indicates that the case is completely outside the deployment area and 0 indicates that the case is completely inside the deployment area. This methodology is then implemented using the following algorithm:

1. Starting in 2012 we generate a random sample of 1,000,000 putative controls from the population of 4186 case–controls by sampling with replacement. We extract the 2012 deployment score for the location of the sampled controls. Subsequently:
  - We take the cases that were infected during that year and sum their inverted deployment scores. The resulting summed score gives us our benchmark score.
  - For the 1,000,000 samples, we take the cumulative sum of the scores of the sampled case–controls.
  - We perform 10,000 iterations, where we:
    i. Count how many controls are required until the cumulative score of the sampled controls is greater than the benchmark identified above. This number of controls is our sample index. So, if our benchmark score is 5 and the cumulative sum of our controls is 0, 2, 3, 6, 7, 9, 9, 12, then we take the fourth score of 6 as our sampled score, and the sample index is four.
    ii. We evaluate the values above and below the sample index to determine which is closer to the total of our cases. So from the example above we take the third value of 3 and fourth value of 6. As our benchmark of 5 is nearer to the fourth value than the third, we take the fourth value as our number of controls. Had the benchmark been 5 and the sequence 0, 2, 4, 6, then we select at random between 4 and 6.
    iii. Record how many sampled case–controls were required to match the total of the cases. In the example above this is four.
    iv. From the 1,000,000 samples we remove all values up to and including our sample index and repeat the cumulative sum. So in the example above with the sequence 0, 2, 3, 6, 7, 9, 9, 12, we remove the first four values and subtract 6 from the remainder, so our sample now starts 1, 3, 3, 6.
    v. Repeat i–iv until we have completed 10,000 iterations.
2. Repeat 1 for each subsequent year 2013, 2104…2019.

We analyse this by comparing the number of cases from each year to the numbers of controls that were sampled in each iteration in each year above. In a given year, if more than 95% of iterations had a number of controls that was greater than the number of cases for that year, then it would indicate that there was a significant difference between the number of cases and the random sample. The difference between the median of the samples and the number of cases gives the median number of excess cases.

### Sensitivity analysis

We tested the results using a number of sensitivity analyses:

1. The earlier years of Tiny Target deployments had a smaller extent of deployment and a greater number of cases, so we test the robustness of the results by excluding these years.
2. We adjust the deployment scores using three methodologies. The first increases the scores of 2 and 3 by one point, so the scale is 0, 1, 3, 4. The second is multiplicative by two, so the revised scale is 0, 2, 4, 6. The third is a square transformation, so the scale is 0, 1, 4, 9.
3. We conducted three separate leave-one-out analyses on the deployment impact methodology. In the first instance we repeat the analysis and resulting probability by dropping in turn each case infected between 2011 and 2019. The second analysis takes the ranks of these probabilities and drops first the highest ranked case, then also the second, third, …*n*th ranked case, repeating the analysis after each drop to re-evaluate the remaining probability. The third analysis leaves 1, 2, 3… *n* cases out at random (repeating the sampling 10,000 times for each number of cases that are left out). To account for the variability in this sampling we generate 95% confidence intervals around the resulting mean probability.
4. A further analysis evaluates the resulting probability after adding further cases in the controlled zone (deployment score 3) in 2019.

## Results

The deployment scores increased year-on-year, but the greatest increase was between 2014 and 2015 when the deployment expanded in the core area (Tables 2 and 3, Figs. 3 and 4, Additional file 1: Figures S1–S8, Additional file 2: Figures S9–S17). The deployment zone scores for cases were lower than those for putative controls in all years apart from 2014 (Fig. 5). The decrease was particularly pronounced from 2015 onwards, when the extent of target deployment was at its greatest and mean scores for controls were greatest (Fig. 5).

**Table 2:** The area and number of watersheds under different deployment scores during each year of the intervention

Year	Watershed areas (km^2^)				Number of watersheds			
	Deployment score^a^				Deployment score^a^			
	0	1	2	3	0	1	2	3
2012	20,381	659	335	2	2048	55	29	1
2013	20,249	523	403	201	2032	47	32	22
2014	19,529	937	675	235	1966	83	58	26
2015	16,923	1894	1745	814	1733	174	143	83
2016	16,780	1974	1709	913	1718	181	144	90
2017	15,145	2914	2331	987	1560	276	201	96
2018	14,357	3105	2773	1141	1486	295	240	112
2019	14,357	3105	2773	1141	1486	295	240	112

^a^Deployment scores 0 = totally outside the controlled area, 1 = outside but neighbouring the controlled area, 2 = inside the controlled area, but bordering non-controlled areas, 3 = totally inside the controlled area

**Table 3:** The number of case–controls (denominator) in each deployment score category by year (left) and (right) the number of cases infected in each year by the case’s deployment score category of that year

Year	Denominator				Case data			
	Deployment score^a^				Deployment score^a^			
	0	1	2	3	0	1	2	3
2012	3528	349	309	0	10	0	1	0
2013	3453	157	304	272	11	0	0	0
2014	2012	434	1422	318	3	0	0	2
2015	997	340	1955	894	3	0	0	1
2016	968	359	1808	1051	0	1	0	0
2017	651	458	1833	1244	0	0	1	0
2018	520	502	1810	1354	1	1	0	0
2019	505	514	1810	1357	1	0	0	0

^a^Deployment scores 0 = totally outside the controlled area, 1 = outside but neighbouring the controlled area, 2 = inside the controlled area, but bordering non-controlled areas, 3 = totally inside the controlled area

**Fig. 4:**
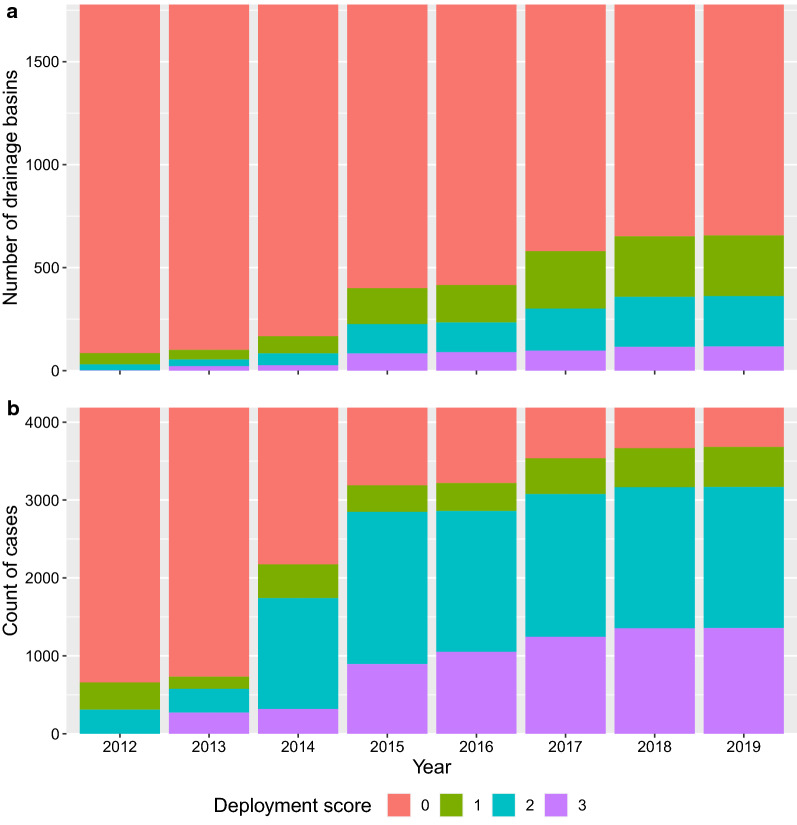
Bar chart of the watershed deployment scores per year for watersheds that are within the deployment districts (**a**). The total number of cases between 2000 and 2020 (*n* = 4187), broken down by the deployment zone scores for each year of intervention (**b**)

**Fig. 5:**
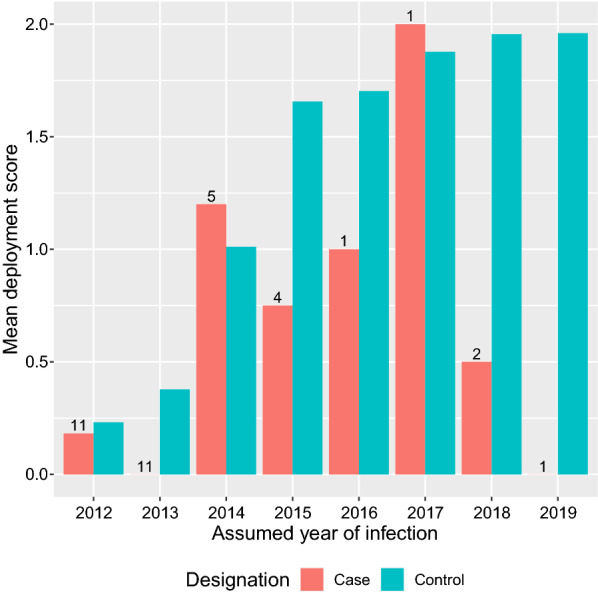
Bar chart of the mean deployment scores for cases and case–controls against the year in which the cases were assumed to have been infected. The numbers above the case bars represent the number of cases assumed to be infected that year. There were 4186 controls in each year

**Fig. 6:**
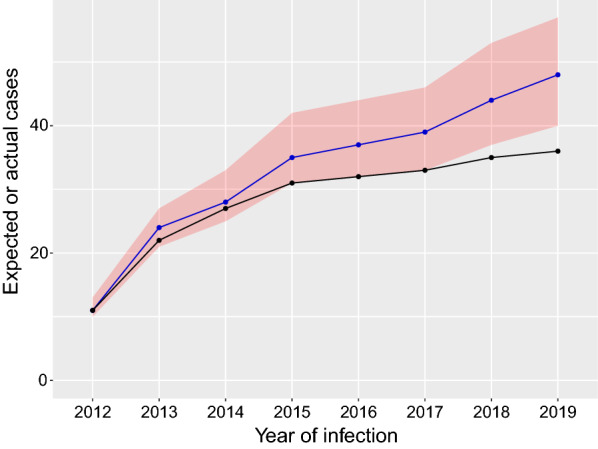
Line chart of the cumulative number of reported cases (black line) and modelled case numbers (blue line). The blue line represents the cumulative number of cases that were modelled without vector control and the red ribbon the 95% confidence around these cases. The black line is the observed number of cases by infection date

Taking the sum of the results over the entire period, the mean sampled case–control deployment score is 1.86× that of the deployment scores for cases infected between 2012 and 2019, and in only 0.7% of iterations were the sampled control deployment scores lower than those for the cases (Table 4 row 1). Furthermore, when we adjust the length of time that we consider, changing the duration from 2012 to 2019 by removing the first year each time, the results remain the same; thus it is insensitive to the period that we analyse (Table 4 rows 2–4). In the analysis of cases prevented, the model shows that by 2019 there would have been a median of 48 infected cases in the focus [95% confidence intervals (CIs) 40–57] if no Tiny Targets had been deployed, whilst there were 36 observed cases (Fig. 6). Our analysis therefore suggests that Tiny Targets prevented 12 cases of gHAT between 2012 and 2019.

**Table 4:** Summary of the statistical model results for different truncations to the study periods

Year range (year of infection)	Total cases in the focus	Case score	Mean case–control score	Case–control score/case score	Proportion of iterations case scores > case–control scores
2012–2019	36	15	27.8	1.86	0.007
2013–2019	25	13	25.2	1.94	0.006
2014–2019	14	13	21.1	1.62	0.027
2015–2019	9	7	16.0	2.29	0.004

Results are summed across the study years

### Sensitivity analysis

The results in Table 2 from 2012–2019 are insensitive to the scoring system that is used (Table 5).

**Table 5:** Sensitivity analysis of different scoring systems and the resulting probabilities

Deployment scores	Probability
Baseline: (Outside = 0; Out–In = 1; In–Out = 2; Inside = 3)	0.007
S1 (additive): (Outside = 0; Out–In = 1; In–Out = 4; Inside = 5)	0.004
S2 (multiplicative): (Outside = 0; Out–In = 2; In–Out = 4; Inside = 6)	0.007
S3 (non-linear): (Outside = 0; Out–In = 1;In–Out = 4; Inside = 9)	0.003

**Fig. 7:**
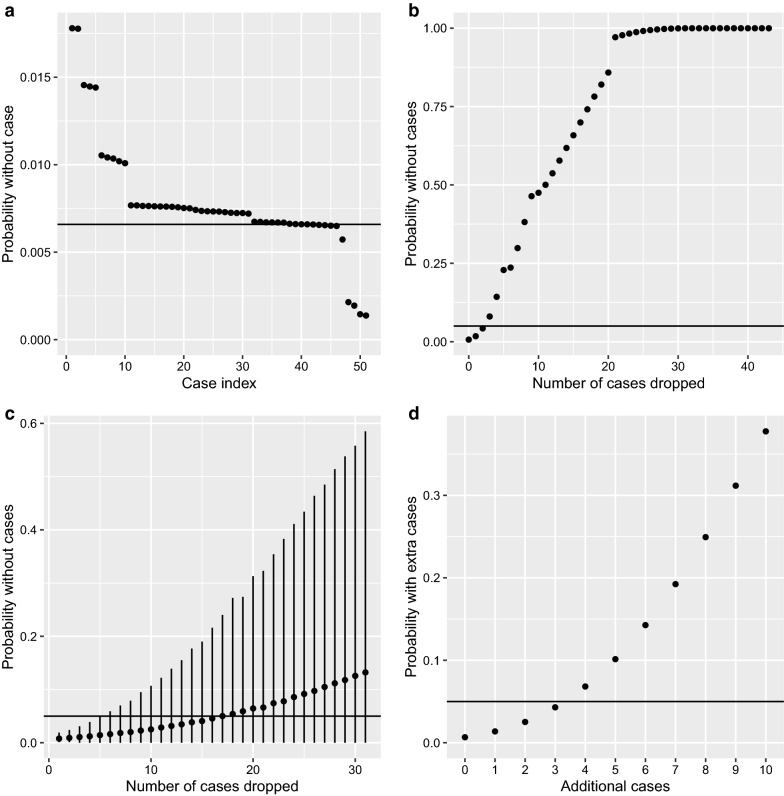
Results of sensitivity analyses. **a** The remaining probability when one case at a time is dropped out; the cases are ranked and the black line shows that basic *P*-value. **b** Starting with the highest ranked case, the resulting *P*-value when no cases are dropped, the top ranked case, the top two ranked, the *n*th ranked cases are dropped. **c** The resulting probability after randomly removing *n* cases; the points are the mean probability and lines 95% confidence intervals. **d** The impacts of adding additional cases to the controlled zone (score 3) in 2019

The leave-one-out analysis showed a strong influence of certain cases and specifically those that were infected outside the controlled zones and infected more recently (Fig. 7). Consequently, if we remove the three most influential cases (infected in 2019, 2018 and 2015) then our result becomes non-significant (Fig. 7). If cases are removed at random, then it is necessary to remove a greater number of cases to move the probability above 0.05 (Fig. 7c).

## Discussion

Tiny Targets are a technology that has been demonstrated to be effective at reducing the numbers of riverine tsetse [9]. However, the purpose of this technology is to control numbers of cases rather than to eradicate the vector, and this has been demonstrated for hyperendemic foci [17] and high-incidence riverine swamp ecosystems [12] but not previously in a large low-incidence (< 1 case/100,000) riverine ecosystem as found in Uganda. This is further complicated by the delay between the deployment of the technology and realisation of any impact on case numbers due to the chronic nature of gHAT. As Tiny Target deployments are being carried out in a greater number of foci in different agro-ecological settings, it is important to establish whether Tiny Targets have an impact on gHAT case numbers in different ecosystems.

The strategy in Uganda has not been for blanket coverage of vector control over the entire area, but rather control that is targeted at those areas with the greatest numbers of cases. Hence, the extent of vector control increased, but never covered the entire area, or the entirety of any district (Tables 2 and 3, Additional file 1: Figures S1–S8). These analyses show that despite the limited extent of vector control, a substantial reduction in case incidence can be achieved.

In Uganda the decline in numbers of HAT cases had been ongoing for several years prior to the commencement of vector control which was associated with active screening campaigns during the decade prior to the commencement of vector control [25, 27]. By reducing transmission, the implementation of Tiny Targets has increased the rate of decline and thus accelerated progress towards elimination. Based on data through the end of 2018, Uganda would be in a position of meeting the gHAT elimination criteria [1], and this low level was maintained in 2019, with only two cases.

The deployment in Uganda presents a number of specific challenges:

1. There was no clear definition of the area of intervention with vector control. Arbitrary boundaries have been drawn for illustrative purposes, but these are not grounded in the epidemiology or entomology of the disease or its vectors. Additionally, whilst an intervention area was defined, there were some variations in the extent of the deployment for certain time points, and this variation between deployments must be accounted for. Here we developed a novel way of defining the area of intervention using the watersheds. This works because Tiny Targets are deployed along the rivers which are the habitat of the tsetse. Thus, by defining the area of intervention in terms of watersheds, we account for the ecology of the tsetse fly. Alternatives include defining buffers or grid cells for the interventions, but watersheds more accurately reflect the tsetse ecology and logistics of the intervention.
2. We do not know the true date of infection of the cases, only the reporting date. To overcome this, we use a conservative approach by setting the date of infection for stage 2 cases as the lower bound on the published range of duration of stage 1 infection. An alternative and less conservative approach would be to estimate the dates of infection from all cases by sampling from the distributions described in Checchi et al. [25].
3. People and vectors are non-stationary but the case locations are point locations, and this does not represent the true extent of potential exposures to infection. We overcome this by basing our analysis on the vector control status of the watershed of the case as well as its neighbours.

However, for these analyses, Uganda also has a number of advantages over other geographies:

1. It has one of the longest histories of Tiny Target deployment, with the first deployments at the end of 2011.
2. In the controlled areas the deployment is complete (every permanent river is deployed).
3. There have been no gross interruptions of deployment.

We have shown that there were fewer cases than would have been expected inside the intervention area and that a median of 12 cases are estimated to have been averted by the vector control intervention. There have been other interventions in this area running concurrently, not least a programme of enhanced passive screening which started in 2013 and had different coverages over the period in question [7]. However, the broad coverage was the entire area, and the programme did not result in an increase in detection of cases which might be expected when screening is enhanced. A similar passive screening programme in a HAT endemic area of the DRC with no vector control did result in an increase in numbers of cases detected [28]. Additionally, there was an active screening campaign in the vector control area which did not detect any HAT cases [29]. It is still possible that there were areas of localised elimination of HAT transmission due to HAT control activities prior to 2012, and that some of these pockets could have coincided with the Tiny Targets deployment. However, due to the extent of the Tiny Targets deployment, this impact would be minimal.

Sensitivity analysis shows that it would be necessary to either remove three very specifically selected cases or add four targeted cases in order to push the probability above 0.05. As there is underreporting of gHAT, the underreporting would need to be biased towards areas that have Tiny Targets. However, the areas that have had the greatest coverage of enhanced passive screening have coincided with the Tiny Target deployments, so we might expect that underreporting would be lower in the controlled areas.

This study does not have the power of a randomised controlled trial but it does add further evidence of the impact of VC on HAT. Whilst the impact of Tiny Targets on case numbers looks modest, the benefits of cessation of transmission must be considered against the other strategies for breaking transmission in a population of over 2 million, or the costs that would be amassed if disease were reintroduced. Consequently, we see this as a valuable tool in ensuring sustained elimination of gHAT, as it is a mechanism for ensuring that the cycle of transmission is broken in a setting where it could be challenging to screen and break transmission using only medical activities in a large and changing population. This could be compounded if there were latent infections of gHAT in the focus, thus preventing transmission from the latent infections [30]. The costs of using Tiny Targets to control tsetse in Uganda have been estimated [11] but not in the context of achieving the elimination of gHAT. These analyses are being carried out by the Human African Trypanosomiasis Modelling and Economic Predictions for Policy (HAT MEPP) programme [31, 32], and will include an evaluation of the relative contribution of Tiny Targets to the elimination goal.

The balance between screening activities and the impacts of vector control as estimated here must be further evaluated using economic analysis to estimate the relative cost-effectiveness of vector control as an intervention.

## Conclusions

These analyses have demonstrated and quantified a clear impact of the Tiny Targets programme on the incidence of gHAT cases which will contribute to the elimination of gHAT in this focus. Whilst the reduction in case numbers appears modest, it makes a critical contribution to elimination in a population too large for systematic mass screening and that has a large transboundary flow of people from neighbouring South Sudan.

## Supplementary Information

**Additional file 1: Figures S1–S8.** Maps showing the vector control intervention in each year from 2012 to 2019. Watersheds were derived from HydroSHEDS created from the NASA SRTM1 DEM using ESRI ArcGIS 10.5.

**Additional file 2: Figures S9–S17.** Maps showing the vector control intervention in each year from 2012 to 2019 with cases overlain. The first figure shows just the baseline cases (2000–2020). Watersheds were derived from HydroSHEDS created from the NASA SRTM1 DEM using ESRI ArcGIS 10.5.

## Data Availability

The data sets on Tiny Target deployments used during the current study are available from the corresponding author on reasonable request. Data on HAT incidence are partially based on the WHO HAT Atlas and are available by application to WHO.

## Abbreviations

CI: Confidence interval
DEM: Digital elevation model
DRC: Democratic Republic of the Congo
HAT: Human African trypanosomiasis
gHAT: Gambian human African trypanosomiasis
rHAT: Rhodesian human African trypanosomiasis
SRTM: Shuttle Radar Topography Mission
