# Supplementary figures and images for: Estimating the impact of Tiny Targets in reducing the incidence of Gambian sleeping sickness in the North-west Uganda focus
Source: Parasit Vectors. 2021 Aug 18;14:410. doi: 10.1186/s13071-021-04889-x (PMC8371857; doi:10.1186/s13071-021-04889-x)

Fig. S1

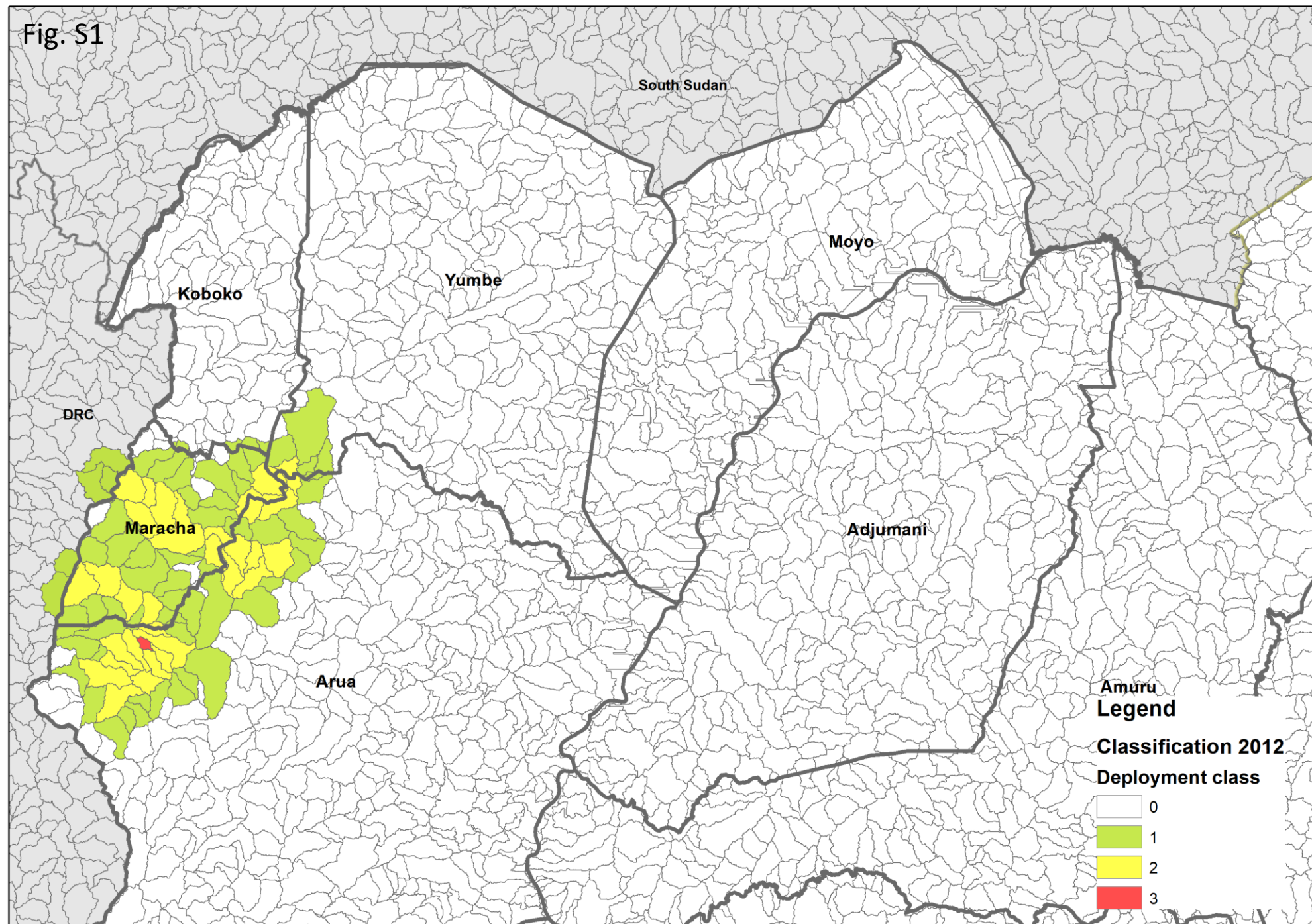

Fig. S2

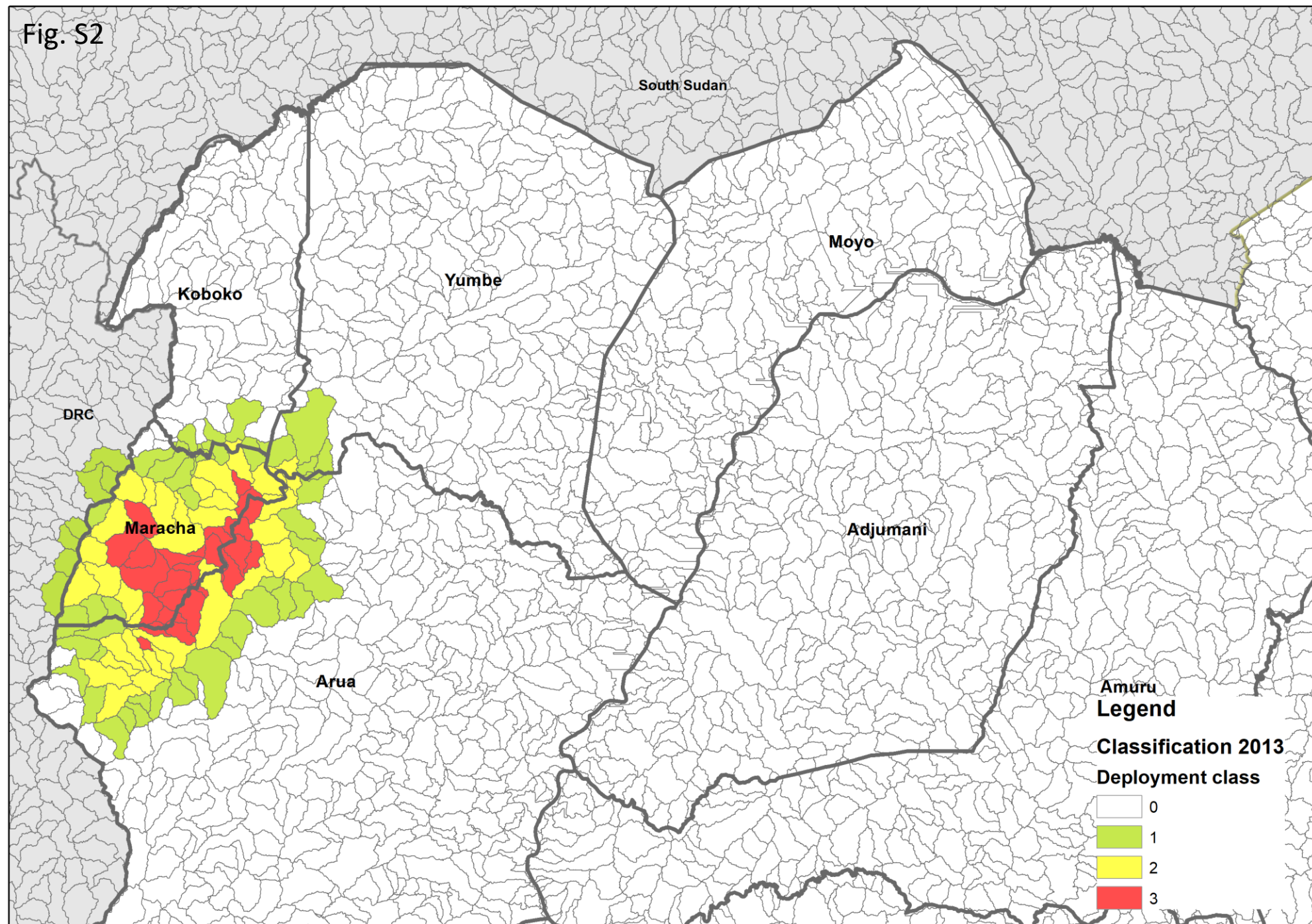

Fig. S3

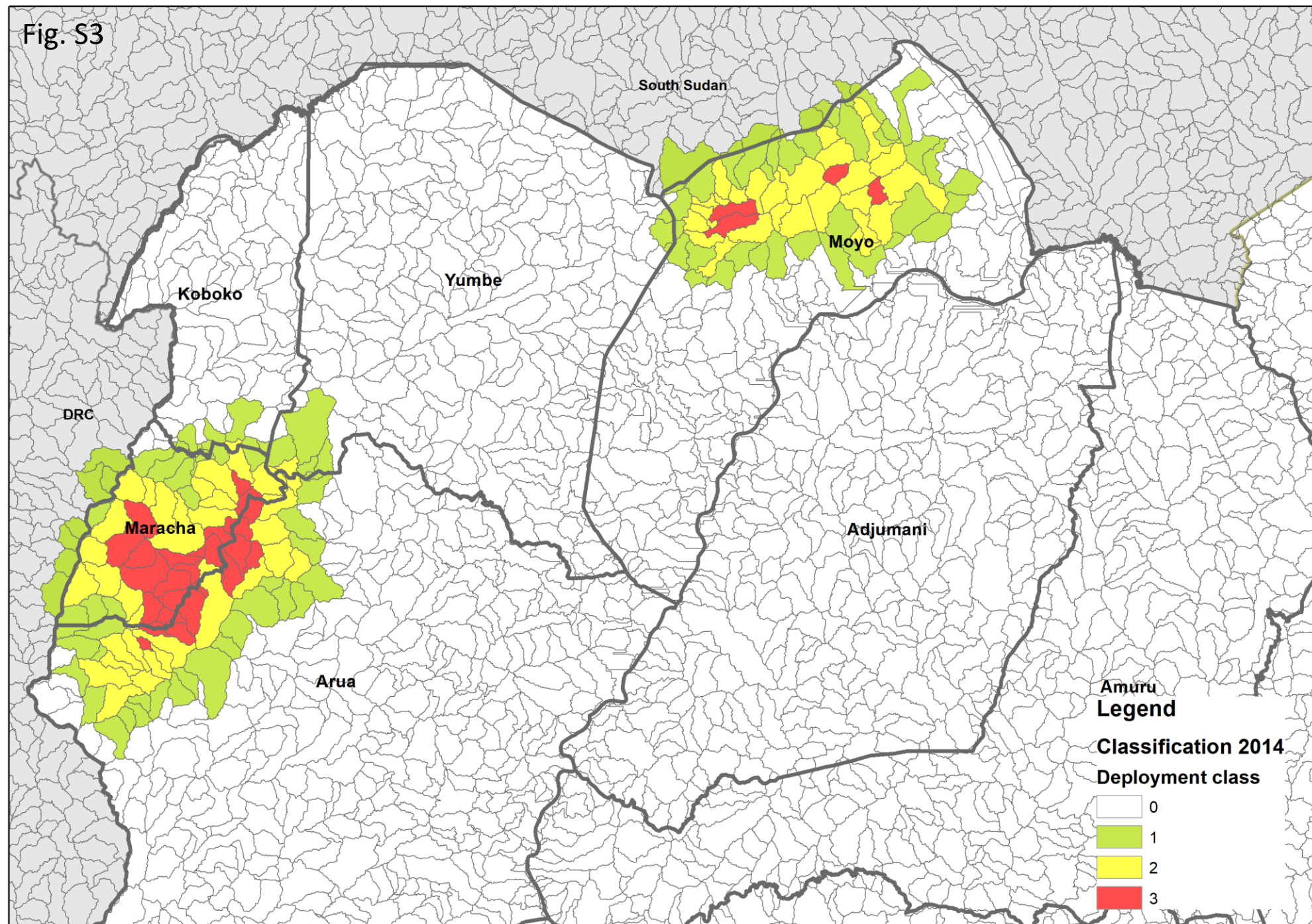

Fig. S4

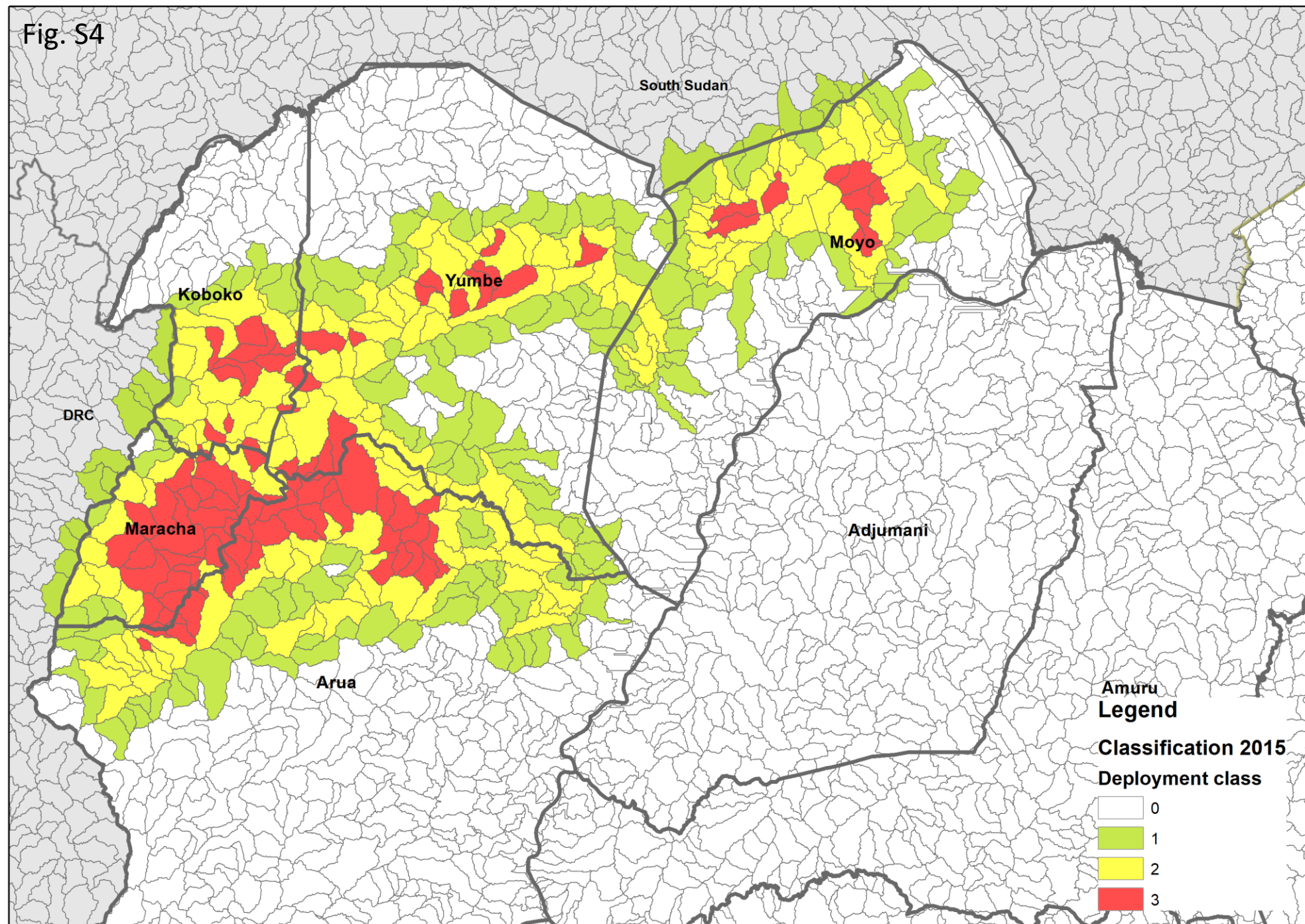

Fig. S5

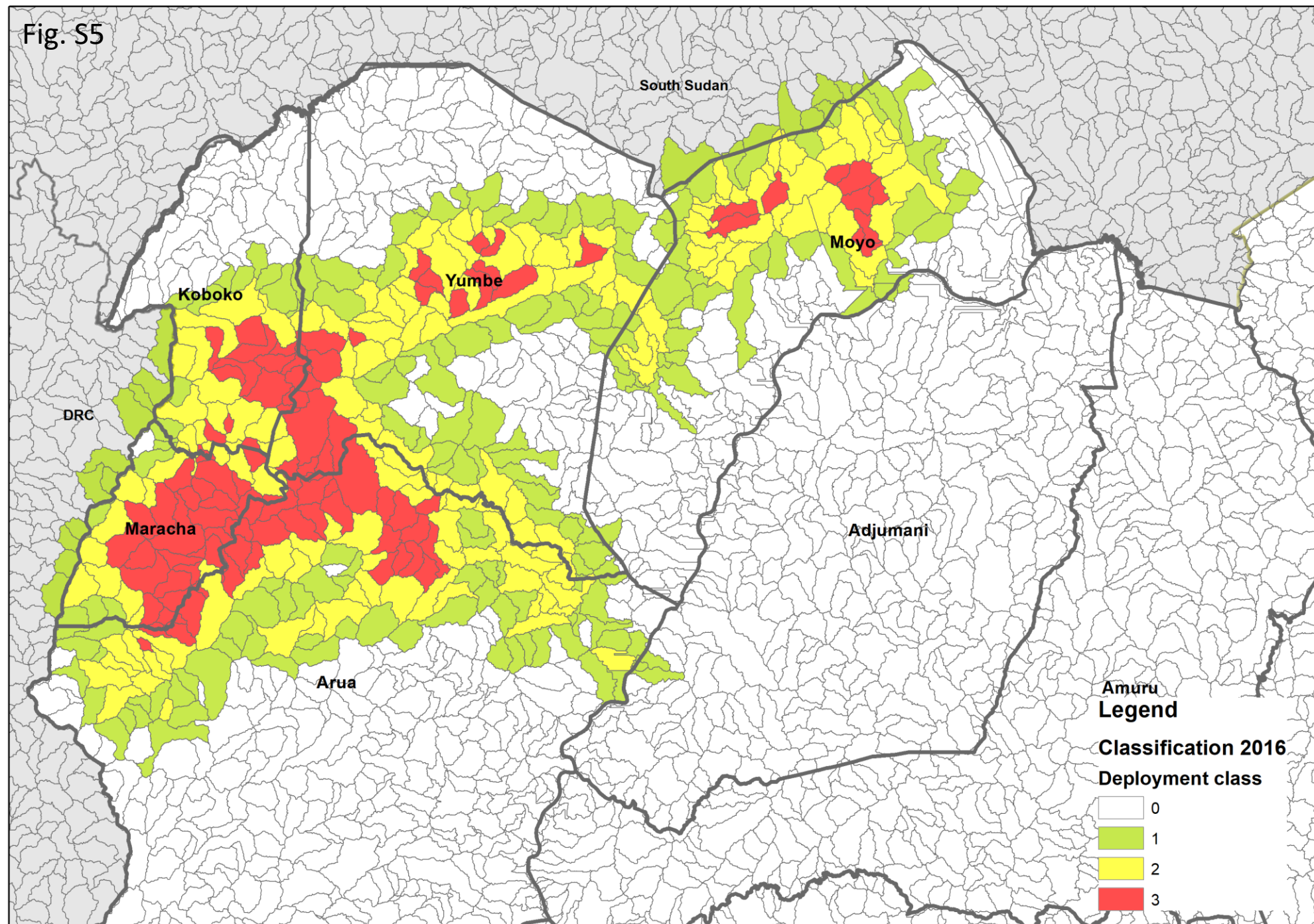

Fig. S6

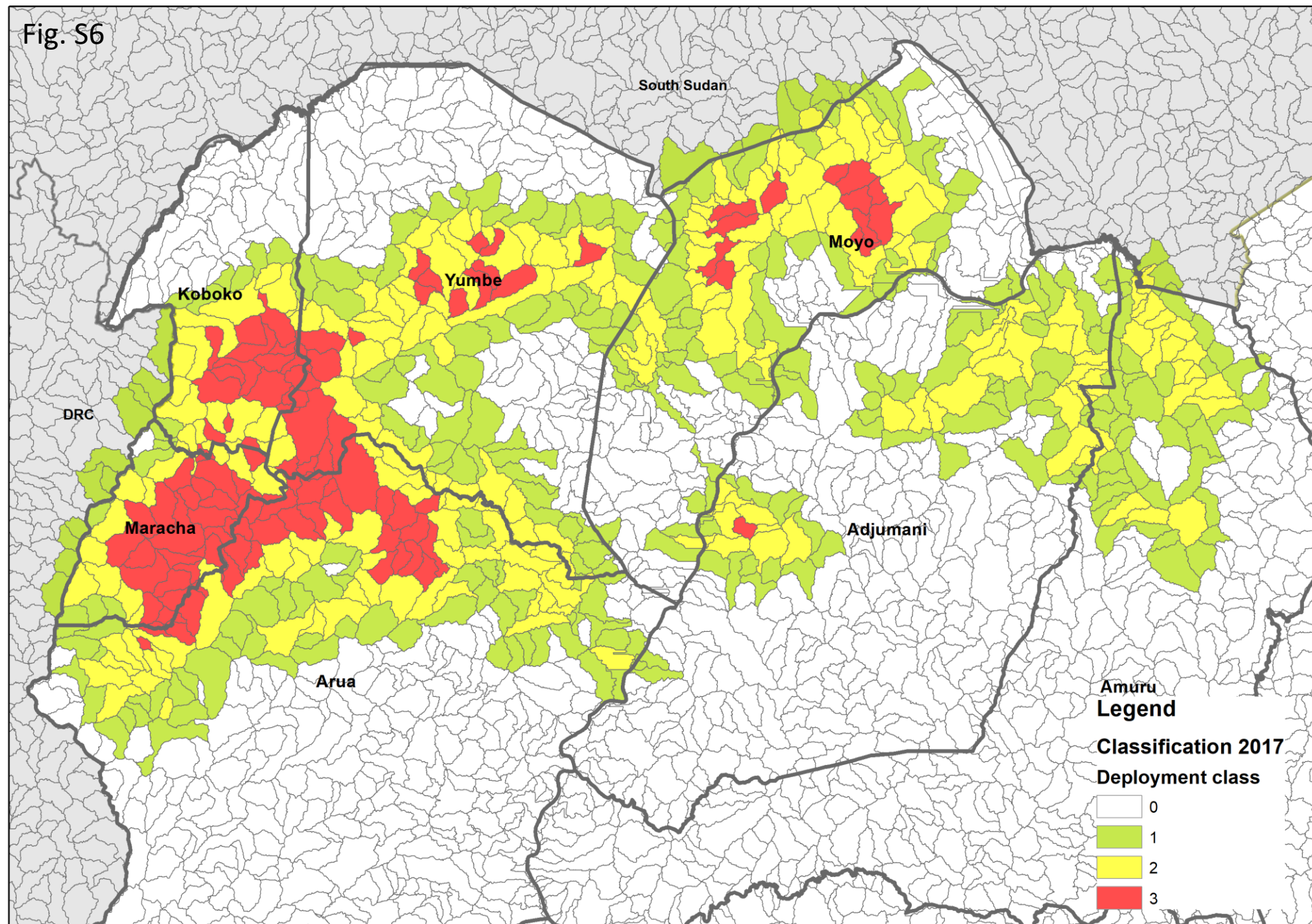

Fig. S7

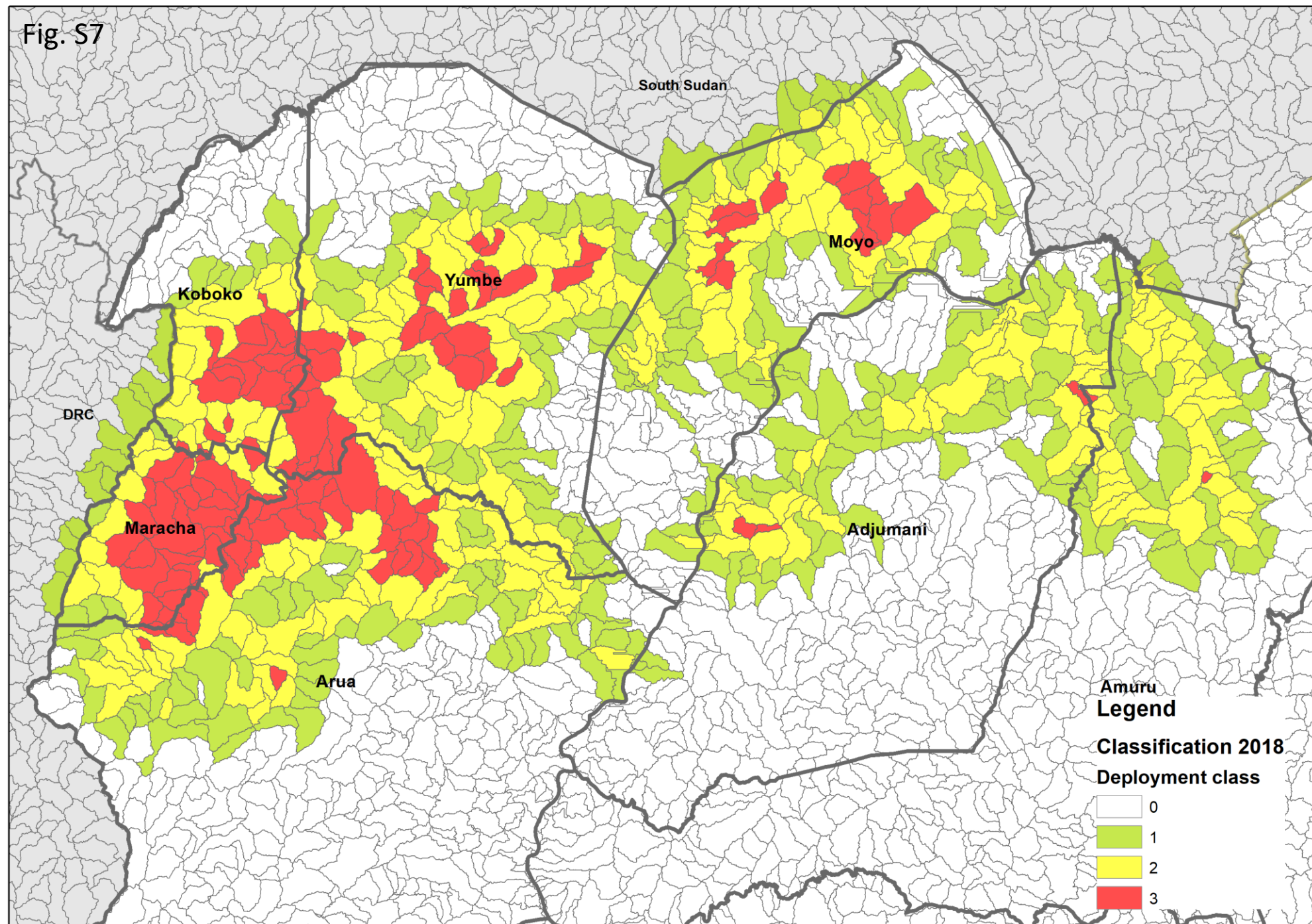

Fig. S8

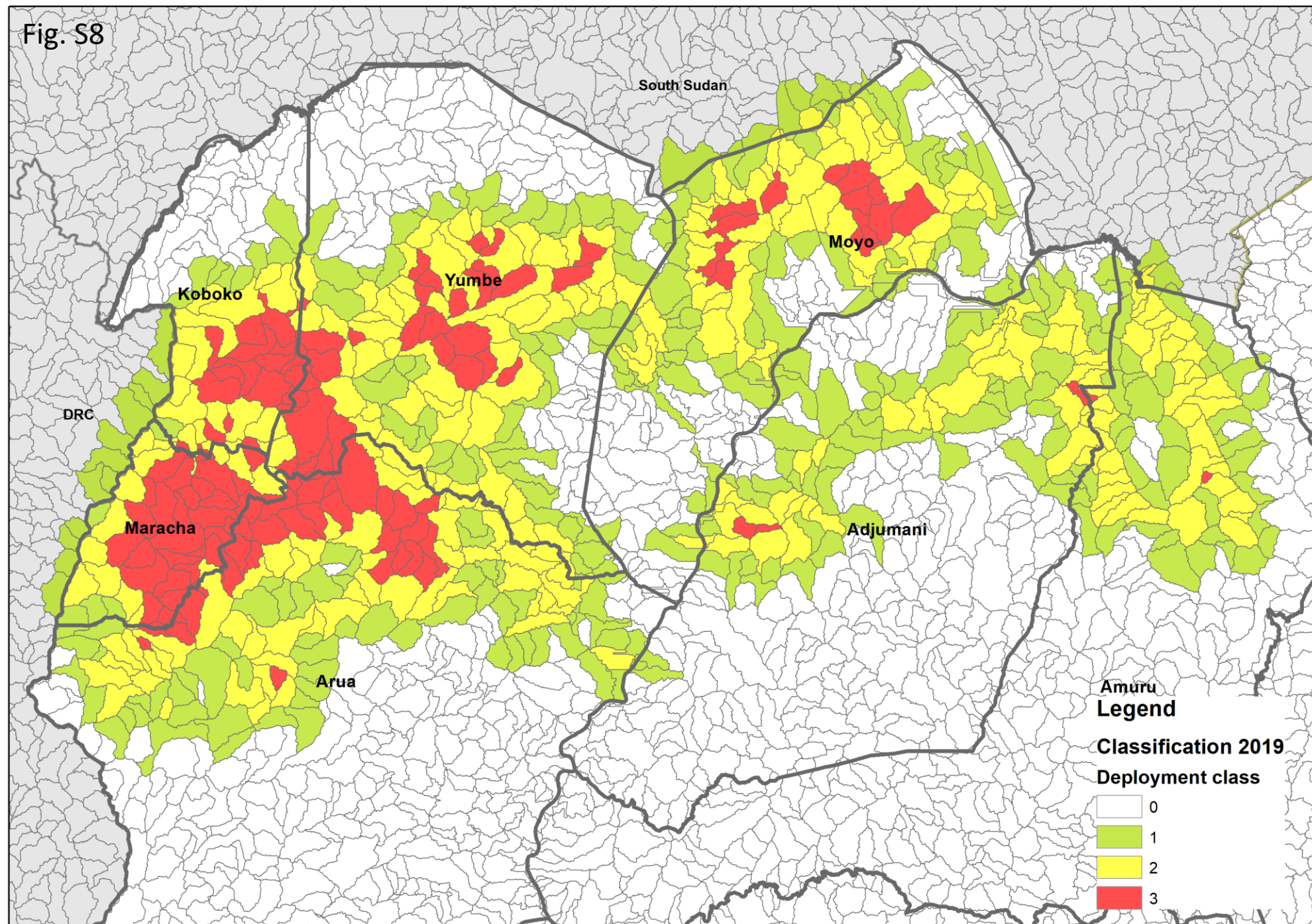

Supplement: Supplementary file 1 — Additional file 1: Figures S1–S8. Maps showing the vector control intervention in each year from 2012 to 2019. Watersheds were derived from HydroSHEDS created from the NASA SRTM1 DEM using ESRI ArcGIS 10.5. [file 13071_2021_4889_MOESM1_ESM.pdf]

Fig. S9

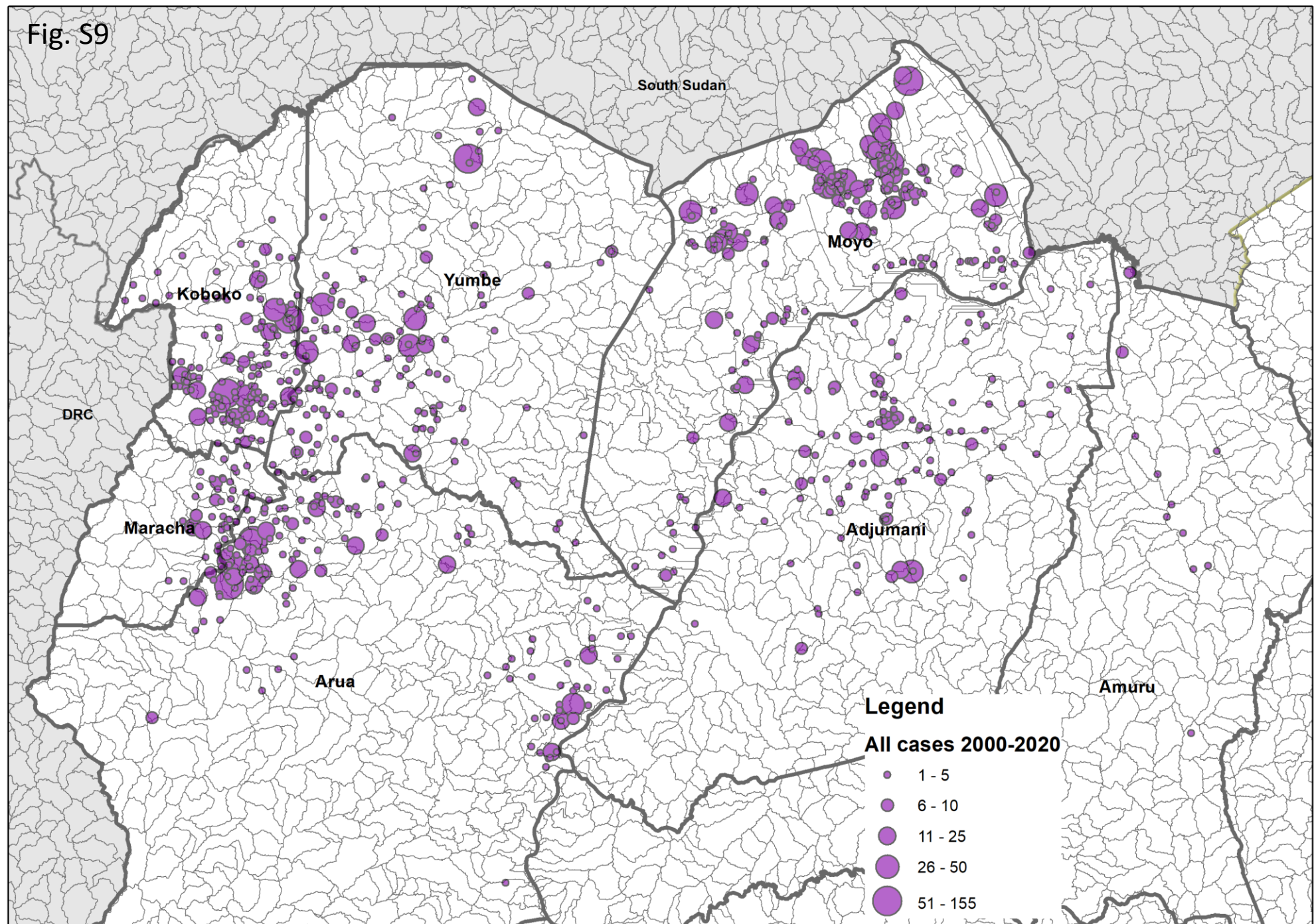

Fig. S10

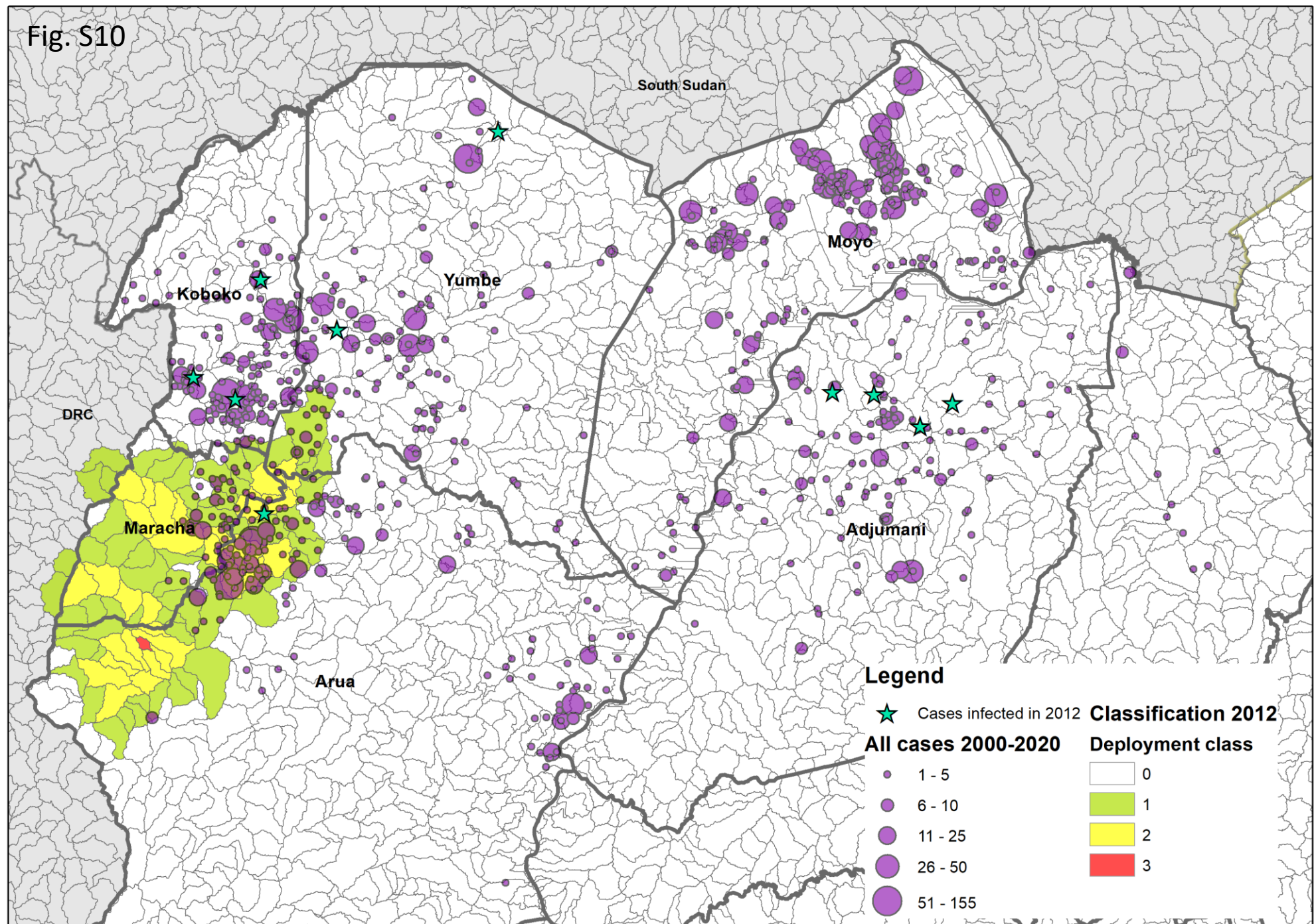

Fig. S11

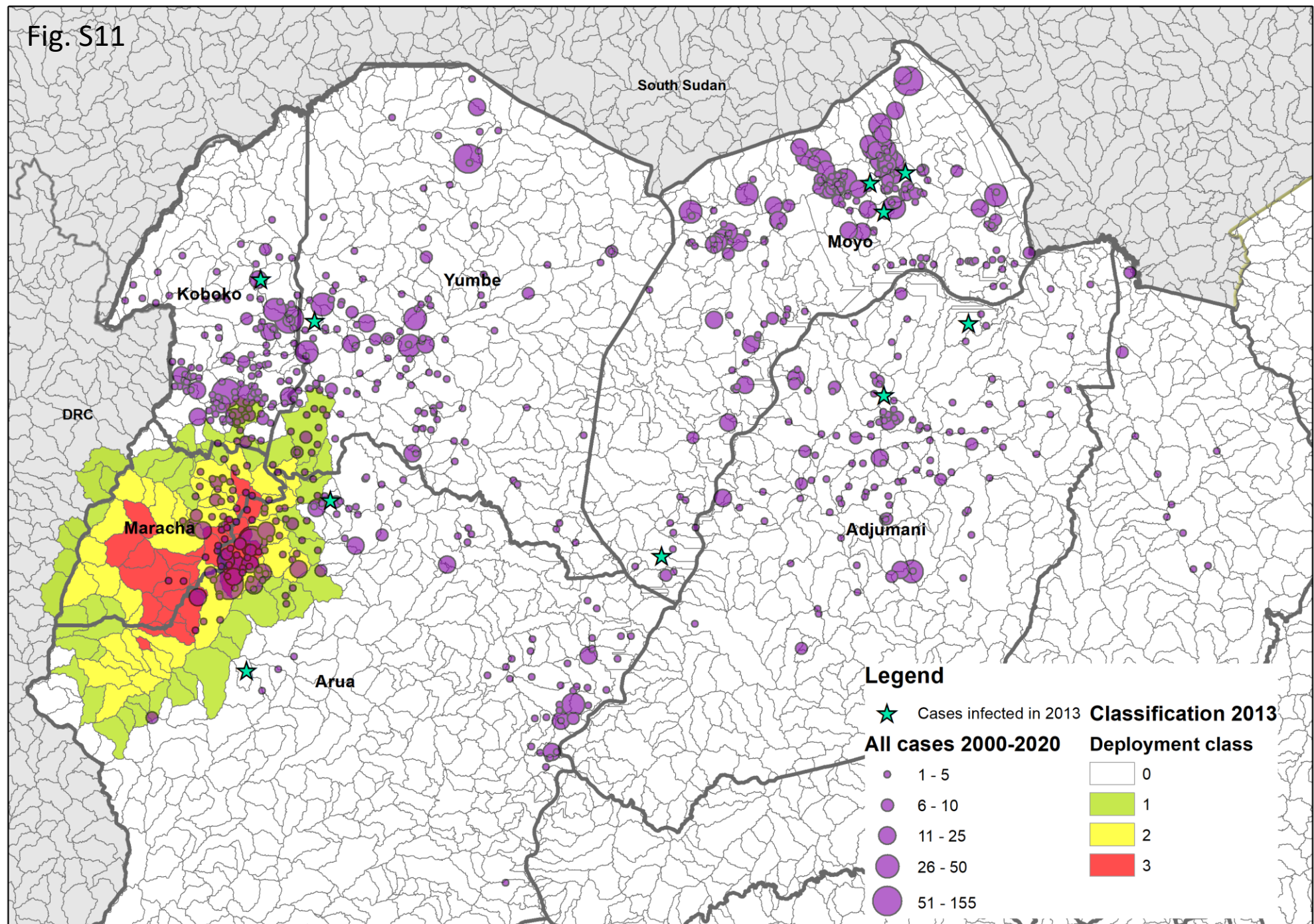

Fig. S12

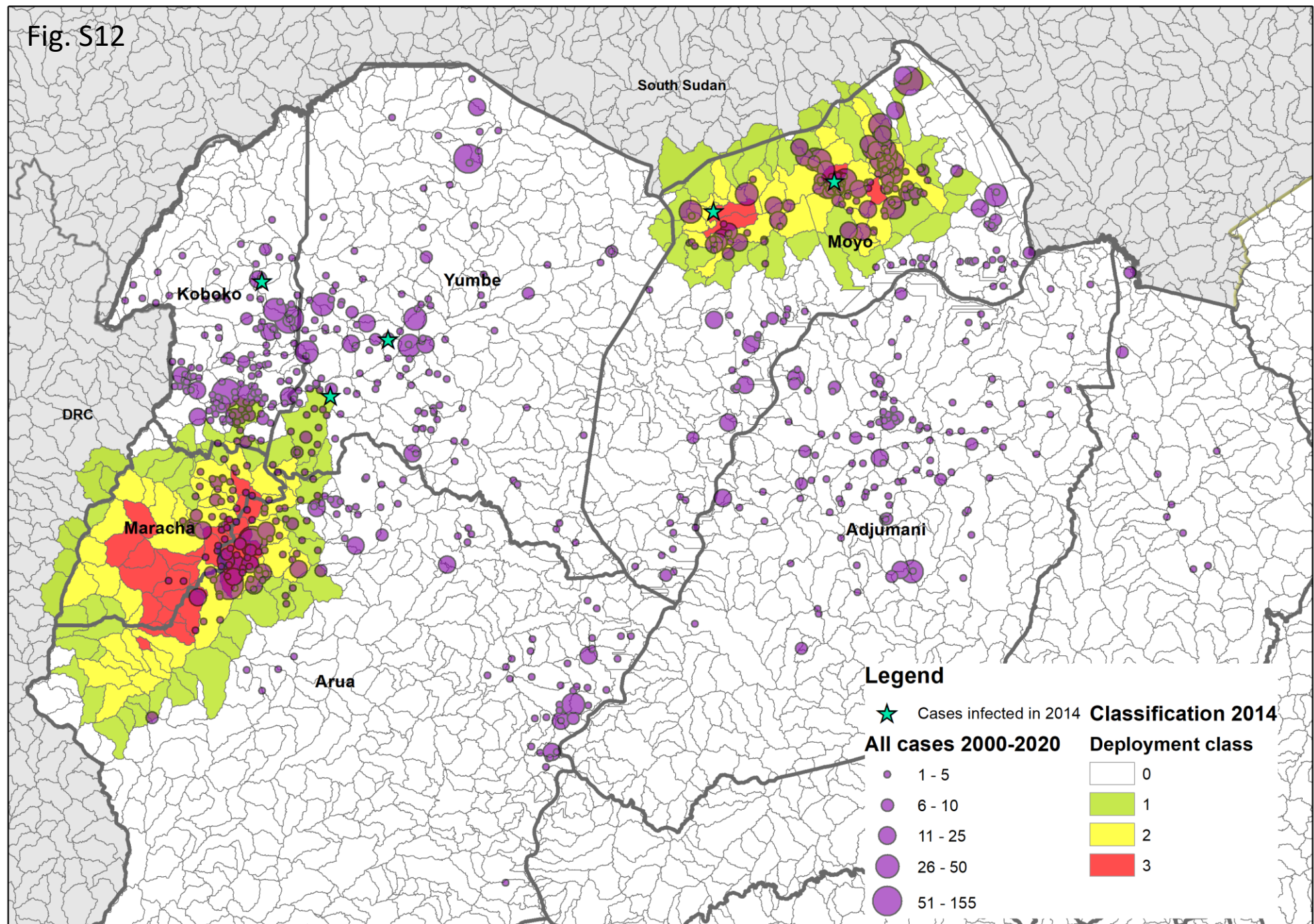

Fig. S13

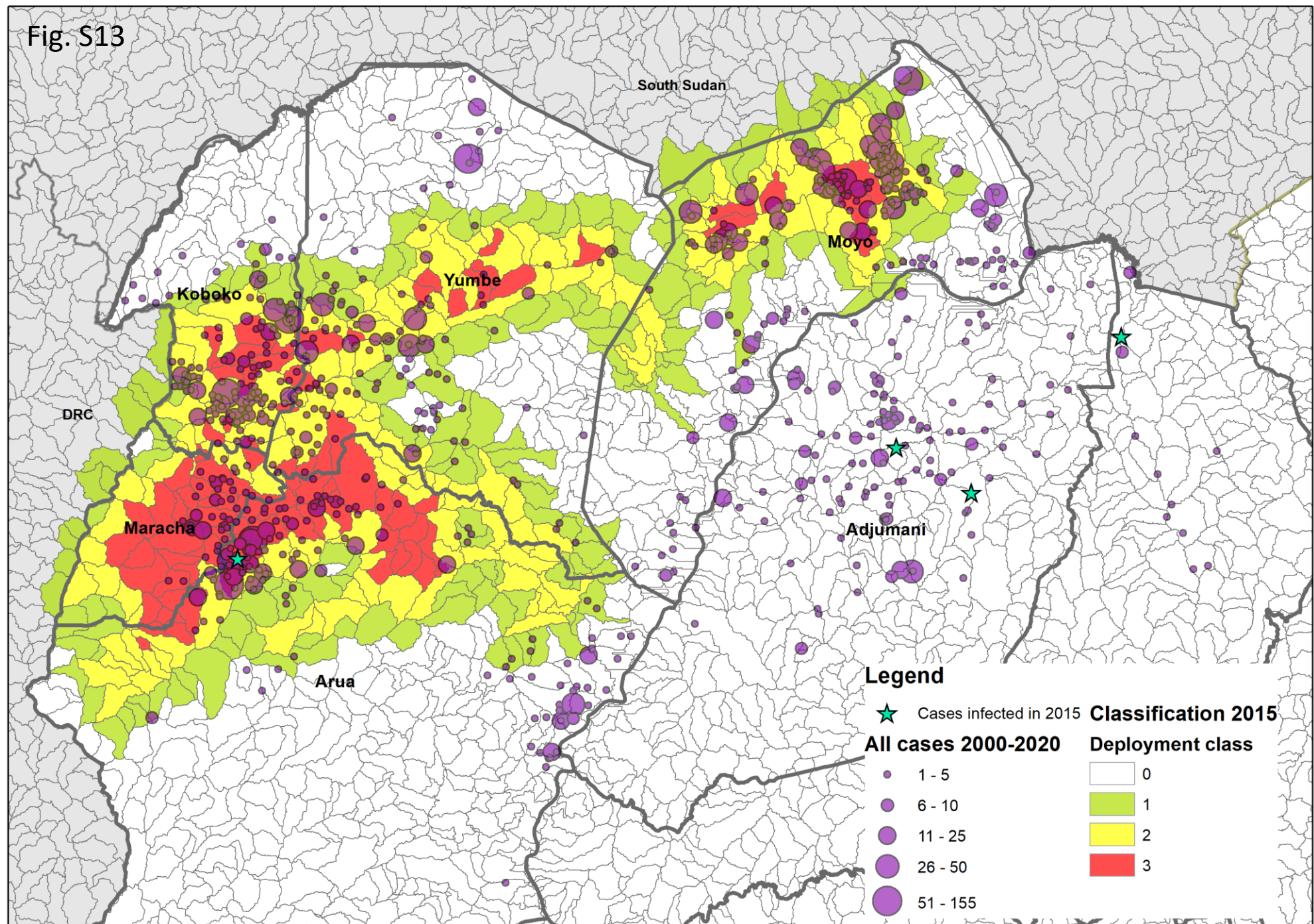

Fig. S14

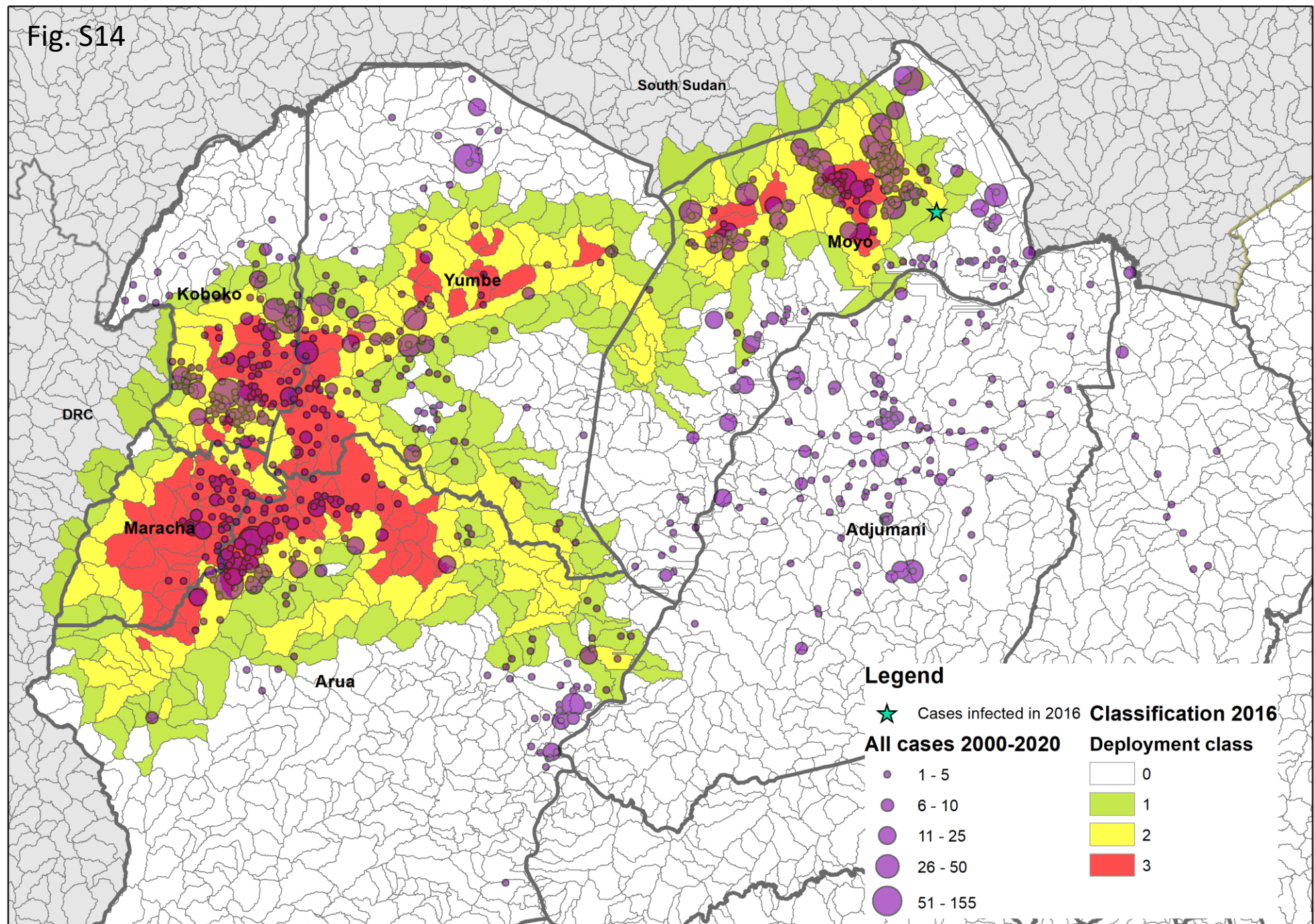

Fig. S15

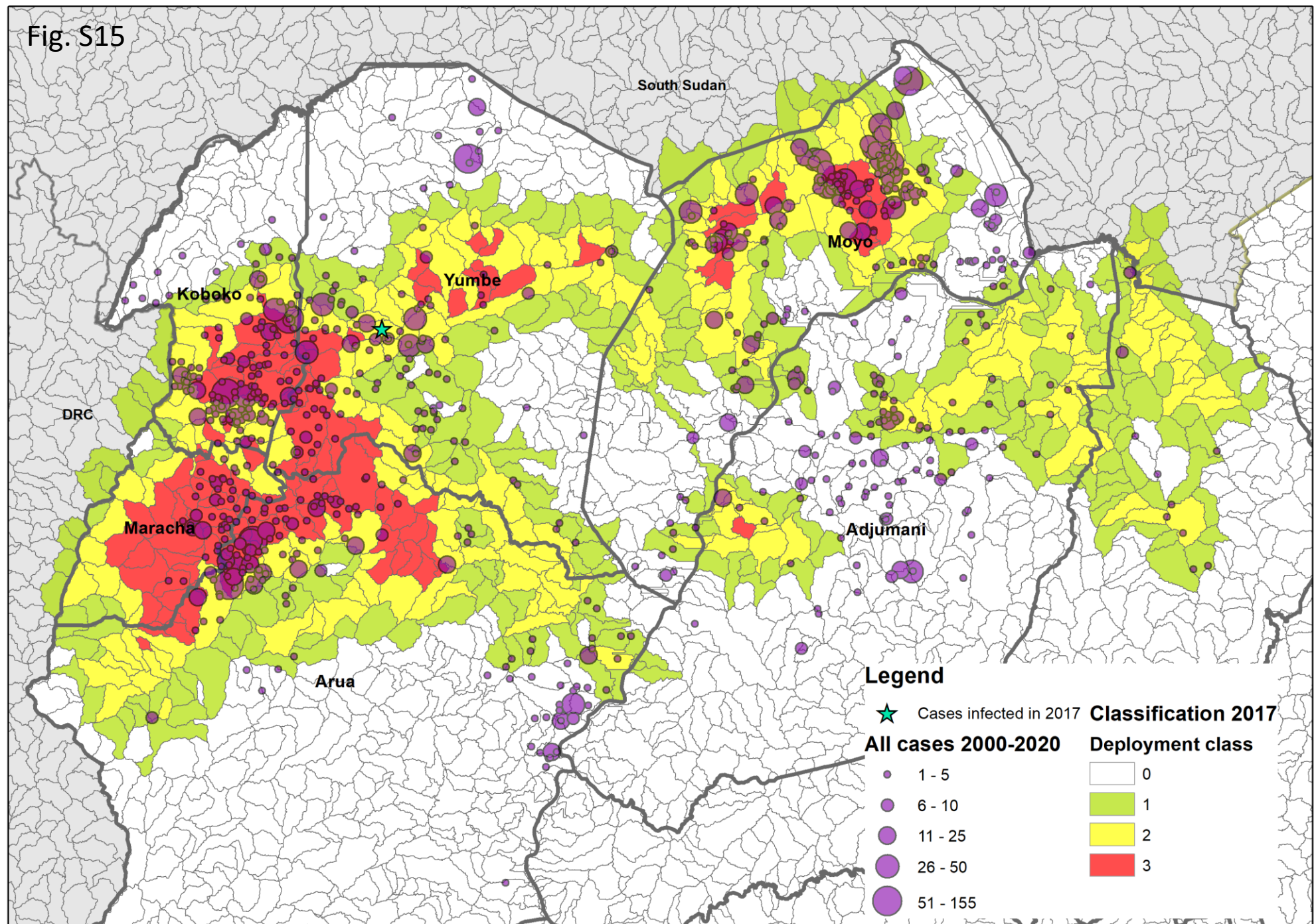

Fig. S16

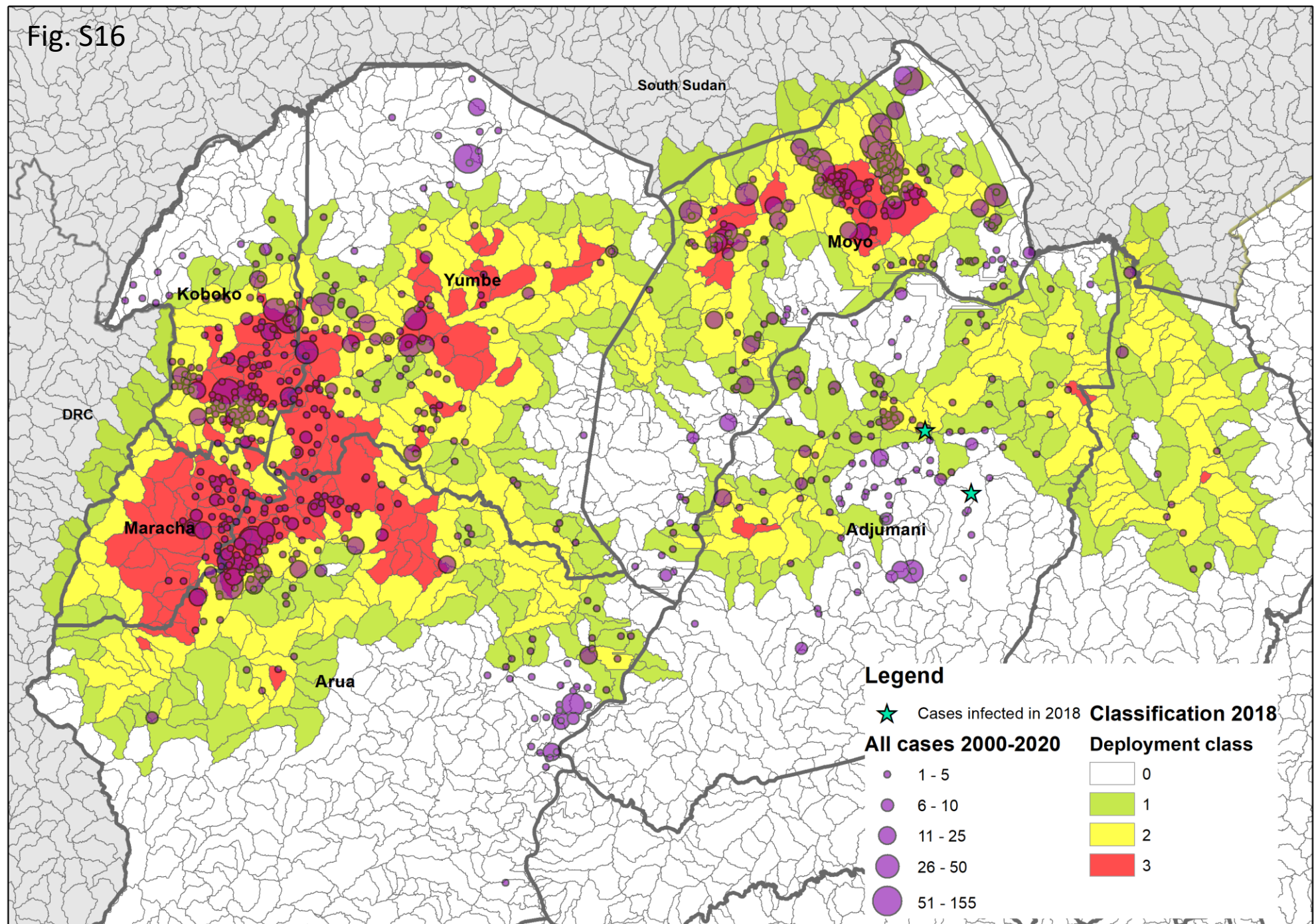

Fig. S17

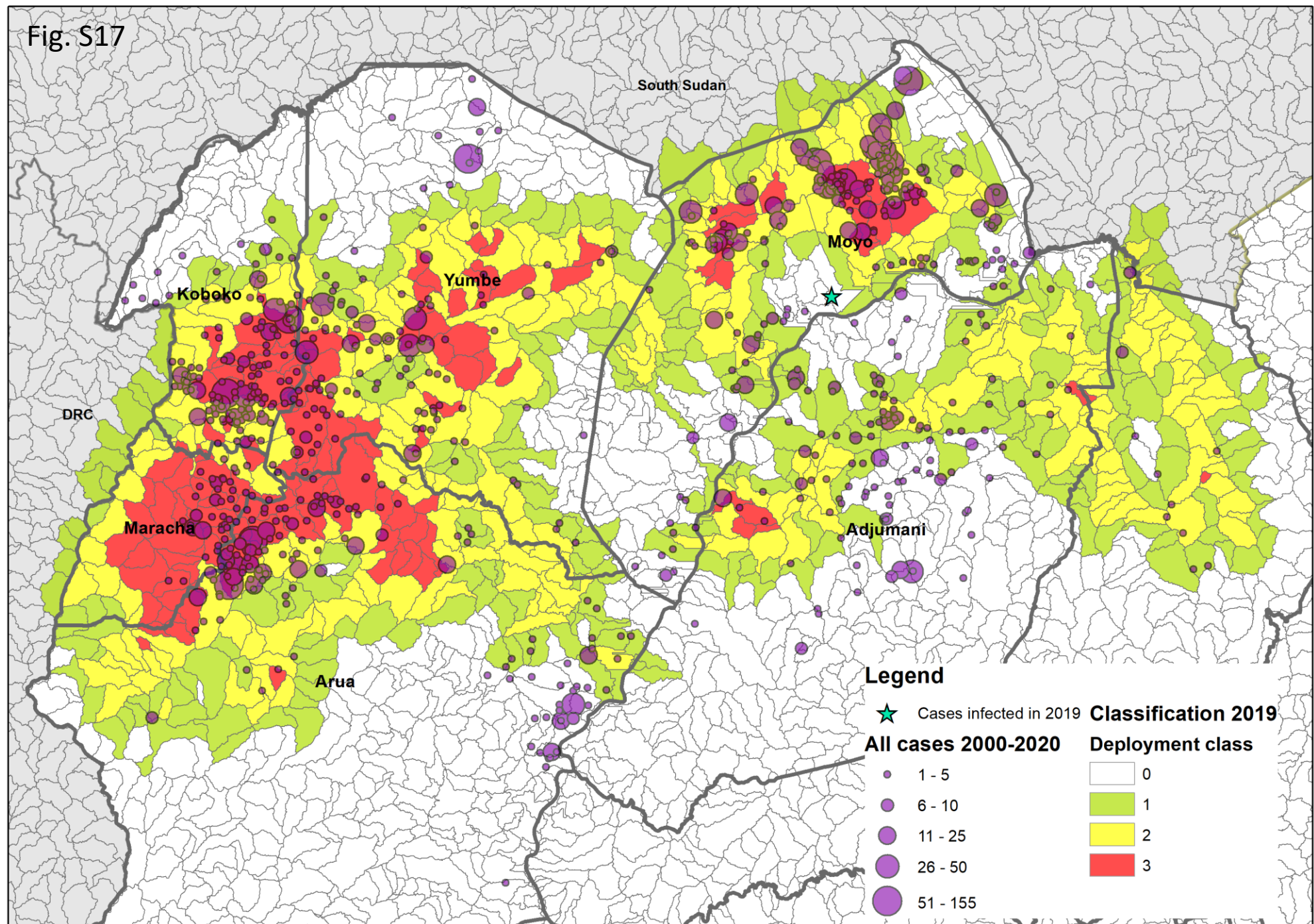

Supplement: Supplementary file 2 — Additional file 2: Figures S9–S17. Maps showing the vector control intervention in each year from 2012 to 2019 with cases overlain. The first figure shows just the baseline cases (2000–2020). Watersheds were derived from HydroSHEDS created from the NASA SRTM1 DEM using ESRI ArcGIS 10.5. [file 13071_2021_4889_MOESM2_ESM.pdf]
